# Supplementary material for: Investigation of 8-Aza-7-Deaza Purine Nucleoside Derivatives
Source: Molecules. 2019 Mar 11;24(5):983. doi: 10.3390/molecules24050983 (PMC6429420; doi:10.3390/molecules24050983)
Supplement: Supplementary file 1 [file molecules-24-00983-s001.zip › molecules-460200 proof supple/supplementory materials Dr. An/cpd 7-Crystallographic information/201804211_tables-7.html]

201804211


# 201804211

Table 1 Crystal data and structure refinement for 201804211.

| Identification code | 201804211 |
| Empirical formula | C10H13N5O5 |
| Formula weight | 283.25 |
| Temperature/K | 293(2) |
| Crystal system | orthorhombic |
| Space group | P212121 |
| a/Å | 4.7669(2) |
| b/Å | 11.0409(5) |
| c/Å | 21.6334(9) |
| α/° | 90 |
| β/° | 90 |
| γ/° | 90 |
| Volume/Å3 | 1138.59(8) |
| Z | 4 |
| ρcalcg/cm3 | 1.652 |
| μ/mm‑1 | 1.157 |
| F(000) | 592.0 |
| Crystal size/mm3 | 0.15 × 0.11 × 0.1 |
| Radiation | CuKα (λ = 1.54184) |
| 2Θ range for data collection/° | 8.174 to 134.138 |
| Index ranges | -3 ≤ h ≤ 5, -13 ≤ k ≤ 13, -25 ≤ l ≤ 25 |
| Reflections collected | 4100 |
| Independent reflections | 2032 [Rint = 0.0304, Rsigma = 0.0405] |
| Data/restraints/parameters | 2032/0/184 |
| Goodness-of-fit on F2 | 1.036 |
| Final R indexes [I>=2σ (I)] | R1 = 0.0366, wR2 = 0.0880 |
| Final R indexes [all data] | R1 = 0.0408, wR2 = 0.0924 |
| Largest diff. peak/hole / e Å-3 | 0.14/-0.21 || Flack parameter | 0.2(2) |

Table 2 Fractional Atomic Coordinates (×104) and Equivalent Isotropic Displacement Parameters (Å2×103) for 201804211. Ueq is defined as 1/3 of of the trace of the orthogonalised UIJ tensor.

| Atom | *x* | *y* | *z* | U(eq) |
| C1' | 4663(6) | 2481(2) | 3422.2(13) | 21.7(6) |
| C2 | 832(6) | 5821(3) | 4035.5(14) | 22.7(6) |
| C2' | 7547(6) | 2355(3) | 3124.7(13) | 22.4(6) |
| C3' | 6988(7) | 1333(3) | 2674.1(13) | 25.2(6) |
| C4 | 3543(6) | 4186(2) | 4141.0(13) | 20.0(6) |
| C4' | 5133(7) | 498(3) | 3059.4(14) | 25.5(6) |
| C5 | 4812(6) | 4588(3) | 4682.5(13) | 22.6(6) |
| C5' | 6688(8) | -481(3) | 3405.0(17) | 35.6(8) |
| C6 | 4144(6) | 5762(3) | 4902.0(13) | 23.5(6) |
| C7 | 6793(7) | 3686(3) | 4838.0(14) | 26.0(6) |
| N1 | 2061(6) | 6311(2) | 4554.1(12) | 26.0(6) |
| N3 | 1519(5) | 4751(2) | 3805.2(11) | 22.0(5) |
| N4 | -1135(6) | 6508(2) | 3770.3(13) | 31.8(6) |
| N8 | 6804(6) | 2803(2) | 4434.6(11) | 26.7(6) |
| N9 | 4761(5) | 3109(2) | 4004.7(11) | 22.5(5) |
| O1 | 3700(5) | 1267.1(18) | 3504.8(11) | 28.8(5) |
| O2 | 8443(5) | 3436(2) | 2836.4(10) | 29.5(5) |
| O3 | 5381(5) | 1733(2) | 2162.3(10) | 32.2(5) |
| O4 | 4822(6) | -1309(2) | 3702.3(11) | 40.6(6) |
| O5 | 5239(5) | 6310(2) | 5342.1(10) | 32.2(5) |

Table 3 Anisotropic Displacement Parameters (Å2×103) for 201804211. The Anisotropic displacement factor exponent takes the form: -2π2[h2a\*2U11+2hka\*b\*U12+…].

| Atom | U11 | U22 | U33 | U23 | U13 | U12 |
| C1' | 20.1(14) | 18.8(13) | 26.2(13) | -1.5(11) | -3.2(12) | -0.2(11) |
| C2 | 22.9(14) | 19.0(13) | 26.1(13) | 1.8(11) | 1.8(12) | 0.8(11) |
| C2' | 20.5(15) | 21.9(14) | 24.7(13) | 0.2(12) | -0.9(12) | 0.0(11) |
| C3' | 24.4(15) | 24.2(15) | 27.0(14) | -4.3(12) | -0.9(12) | 5.4(13) |
| C4 | 20.3(13) | 17.1(12) | 22.7(13) | 0.8(10) | 3.3(12) | -1.3(11) |
| C4' | 26.5(16) | 21.5(14) | 28.4(14) | -5.9(11) | -3.5(12) | 2.3(13) |
| C5 | 25.2(15) | 20.0(13) | 22.8(13) | 2.4(11) | 0.2(12) | -2.0(12) |
| C5' | 38.1(19) | 26.2(16) | 42.4(18) | 5.4(14) | 2.6(16) | 7.6(15) |
| C6 | 27.5(15) | 19.7(14) | 23.2(14) | -1.0(11) | 2.5(12) | -4.8(12) |
| C7 | 28.3(15) | 26.2(15) | 23.6(13) | 2.6(12) | -6.5(12) | 0.1(13) |
| N1 | 32.9(14) | 16.6(11) | 28.4(13) | -4.3(10) | 1.0(11) | 3.8(10) |
| N3 | 21.3(12) | 19.6(12) | 25.1(12) | -0.6(9) | -0.7(10) | -0.1(10) |
| N4 | 34.0(15) | 26.0(13) | 35.4(14) | -5.7(11) | -6.7(12) | 9.9(11) |
| N8 | 28.8(13) | 24.6(12) | 26.7(12) | 3.3(10) | -3.7(11) | 4.5(11) |
| N9 | 22.7(12) | 19.2(12) | 25.7(11) | -0.8(9) | -0.6(10) | 4.7(10) |
| O1 | 26.0(11) | 19.2(10) | 41.4(12) | -5.0(9) | 7.7(9) | -2.6(9) |
| O2 | 29.9(11) | 28.8(12) | 29.8(11) | 2.4(9) | -4.3(9) | -7.3(9) |
| O3 | 40.0(13) | 30.5(13) | 26.0(10) | 2.2(9) | -6.9(10) | -5.0(11) |
| O4 | 52.3(16) | 24.8(11) | 44.7(13) | 1.1(10) | 13.3(13) | 6.0(12) |
| O5 | 44.1(13) | 24.2(10) | 28.4(10) | -4.4(9) | -7.2(10) | -4.5(10) |

Table 4 Bond Lengths for 201804211.

| Atom | Atom | Length/Å |  | Atom | Atom | Length/Å |
| C1' | C2' | 1.524(4) |  | C4 | N3 | 1.360(4) |
| C1' | N9 | 1.439(4) |  | C4 | N9 | 1.356(4) |
| C1' | O1 | 1.428(3) |  | C4' | C5' | 1.510(4) |
| C2 | N1 | 1.377(4) |  | C4' | O1 | 1.454(4) |
| C2 | N3 | 1.323(4) |  | C5 | C6 | 1.416(4) |
| C2 | N4 | 1.335(4) |  | C5 | C7 | 1.413(4) |
| C2' | C3' | 1.515(4) |  | C5' | O4 | 1.428(4) |
| C2' | O2 | 1.413(3) |  | C6 | N1 | 1.386(4) |
| C3' | C4' | 1.525(4) |  | C6 | O5 | 1.243(4) |
| C3' | O3 | 1.417(4) |  | C7 | N8 | 1.308(4) |
| C4 | C5 | 1.391(4) |  | N8 | N9 | 1.388(3) |

Table 5 Bond Angles for 201804211.

| Atom | Atom | Atom | Angle/˚ |  | Atom | Atom | Atom | Angle/˚ |
| N9 | C1' | C2' | 112.6(2) |  | O1 | C4' | C5' | 108.7(3) |
| O1 | C1' | C2' | 104.9(2) |  | C4 | C5 | C6 | 118.4(3) |
| O1 | C1' | N9 | 110.7(2) |  | C4 | C5 | C7 | 105.4(3) |
| N3 | C2 | N1 | 123.6(3) |  | C7 | C5 | C6 | 135.7(3) |
| N3 | C2 | N4 | 121.3(3) |  | O4 | C5' | C4' | 112.1(3) |
| N4 | C2 | N1 | 115.2(3) |  | N1 | C6 | C5 | 112.3(3) |
| C3' | C2' | C1' | 100.4(2) |  | O5 | C6 | C5 | 127.4(3) |
| O2 | C2' | C1' | 112.5(2) |  | O5 | C6 | N1 | 120.2(3) |
| O2 | C2' | C3' | 113.5(2) |  | N8 | C7 | C5 | 111.7(3) |
| C2' | C3' | C4' | 101.6(2) |  | C2 | N1 | C6 | 125.1(2) |
| O3 | C3' | C2' | 111.4(2) |  | C2 | N3 | C4 | 112.6(3) |
| O3 | C3' | C4' | 107.6(3) |  | C7 | N8 | N9 | 105.2(2) |
| N3 | C4 | C5 | 127.8(3) |  | C4 | N9 | C1' | 126.8(2) |
| N9 | C4 | C5 | 106.1(3) |  | C4 | N9 | N8 | 111.6(2) |
| N9 | C4 | N3 | 126.2(3) |  | N8 | N9 | C1' | 119.5(2) |
| C5' | C4' | C3' | 114.8(3) |  | C1' | O1 | C4' | 108.3(2) |
| O1 | C4' | C3' | 106.4(2) |  |  |  |  |  |

Table 6 Hydrogen Bonds for 201804211.

| D | H | A | d(D-H)/Å | d(H-A)/Å | d(D-A)/Å | D-H-A/° |
| N1 | H1 | O51 | 0.86 | 1.94 | 2.775(3) | 161.9 |
| N4 | H4A | O32 | 0.86 | 2.15 | 2.869(3) | 140.9 |
| N4 | H4B | O43 | 0.86 | 2.43 | 3.090(4) | 133.5 |
| N4 | H4B | O51 | 0.86 | 2.44 | 3.150(3) | 140.7 |
| O2 | H2 | N34 | 0.82 | 2.13 | 2.941(3) | 172.9 |
| O3 | H3 | O45 | 0.82 | 2.21 | 2.860(3) | 135.7 |
| O4 | H4 | O56 | 0.82 | 2.34 | 3.008(3) | 139.3 |

1-1/2+X,3/2-Y,1-Z; 2-X,1/2+Y,1/2-Z; 3-1+X,1+Y,+Z; 41+X,+Y,+Z; 51-X,1/2+Y,1/2-Z; 6-1/2+X,1/2-Y,1-Z

Table 7 Torsion Angles for 201804211.

| A | B | C | D | Angle/˚ |  | A | B | C | D | Angle/˚ |
| C1' | C2' | C3' | C4' | -40.3(3) |  | N3 | C2 | N1 | C6 | -0.2(5) |
| C1' | C2' | C3' | O3 | 74.0(3) |  | N3 | C4 | C5 | C6 | -5.5(5) |
| C2' | C1' | N9 | C4 | 116.3(3) |  | N3 | C4 | C5 | C7 | -179.0(3) |
| C2' | C1' | N9 | N8 | -45.7(3) |  | N3 | C4 | N9 | C1' | 15.3(5) |
| C2' | C1' | O1 | C4' | -24.2(3) |  | N3 | C4 | N9 | N8 | 178.5(3) |
| C2' | C3' | C4' | C5' | -92.9(3) |  | N4 | C2 | N1 | C6 | -179.9(3) |
| C2' | C3' | C4' | O1 | 27.3(3) |  | N4 | C2 | N3 | C4 | -179.6(3) |
| C3' | C4' | C5' | O4 | -173.7(2) |  | N9 | C1' | C2' | C3' | 161.0(2) |
| C3' | C4' | O1 | C1' | -2.1(3) |  | N9 | C1' | C2' | O2 | -78.0(3) |
| C4 | C5 | C6 | N1 | 5.2(4) |  | N9 | C1' | O1 | C4' | -145.9(2) |
| C4 | C5 | C6 | O5 | -173.2(3) |  | N9 | C4 | C5 | C6 | 173.7(3) |
| C4 | C5 | C7 | N8 | 0.5(4) |  | N9 | C4 | C5 | C7 | 0.2(3) |
| C5 | C4 | N3 | C2 | 2.2(4) |  | N9 | C4 | N3 | C2 | -176.9(3) |
| C5 | C4 | N9 | C1' | -164.0(3) |  | O1 | C1' | C2' | C3' | 40.6(3) |
| C5 | C4 | N9 | N8 | -0.8(3) |  | O1 | C1' | C2' | O2 | 161.5(2) |
| C5 | C6 | N1 | C2 | -2.8(4) |  | O1 | C1' | N9 | C4 | -126.6(3) |
| C5 | C7 | N8 | N9 | -0.9(3) |  | O1 | C1' | N9 | N8 | 71.3(3) |
| C5' | C4' | O1 | C1' | 122.0(3) |  | O1 | C4' | C5' | O4 | 67.4(3) |
| C6 | C5 | C7 | N8 | -171.4(3) |  | O2 | C2' | C3' | C4' | -160.5(2) |
| C7 | C5 | C6 | N1 | 176.3(3) |  | O2 | C2' | C3' | O3 | -46.2(3) |
| C7 | C5 | C6 | O5 | -2.1(6) |  | O3 | C3' | C4' | C5' | 150.0(3) |
| C7 | N8 | N9 | C1' | 165.6(3) |  | O3 | C3' | C4' | O1 | -89.8(3) |
| C7 | N8 | N9 | C4 | 1.0(3) |  | O5 | C6 | N1 | C2 | 175.7(3) |
| N1 | C2 | N3 | C4 | 0.7(4) |  |  |  |  |  |  |

Table 8 Hydrogen Atom Coordinates (Å×104) and Isotropic Displacement Parameters (Å2×103) for 201804211.

| Atom | *x* | *y* | *z* | U(eq) |
| H1' | 3401 | 2909 | 3139 | 26 |
| H2' | 8926 | 2099 | 3434 | 27 |
| H3' | 8724 | 931 | 2544 | 30 |
| H4' | 3735 | 121 | 2789 | 31 |
| H5'A | 7871 | -923 | 3118 | 43 |
| H5'B | 7892 | -111 | 3713 | 43 |
| H7 | 7946 | 3716 | 5184 | 31 |
| H1 | 1492 | 7014 | 4671 | 31 |
| H4A | -1981 | 6257 | 3443 | 38 |
| H4B | -1554 | 7201 | 3926 | 38 |
| H2 | 9277 | 3859 | 3088 | 44 |
| H3 | 6248 | 2261 | 1976 | 48 |
| H4 | 3933 | -952 | 3971 | 61 |

Experimental

Single crystals of C10H13N5O5
[201804211]
were
[].
A suitable crystal was selected and
[]
on a
Xcalibur, Eos, Gemini
diffractometer. The crystal was kept at 293(2) K during data collection.
Using Olex2 [1], the structure was solved with the
ShelXS
[2] structure solution program using
Direct Methods
and refined with the
ShelXL
[3] refinement package using
Least Squares
minimisation.

1. Dolomanov, O.V., Bourhis, L.J., Gildea, R.J, Howard, J.A.K. & Puschmann, H.
   (2009), J. Appl. Cryst. 42, 339-341.
2. Sheldrick, G.M. (2008). Acta Cryst. A64, 112-122.
3. Sheldrick, G.M. (2015). Acta Cryst. C71, 3-8.

Crystal structure determination of
[201804211]

**Crystal Data**
for C10H13N5O5 (*M*=283.25 g/mol):
orthorhombic, space group P212121 (no. 19),
*a* = 4.7669(2) Å, *b* = 11.0409(5) Å, *c* = 21.6334(9) Å,
*V*= 1138.59(8) Å3,
*Z* = 4,
*T* = 293(2) K,
μ(CuKα) = 1.157 mm-1,
*Dcalc* = 1.652 g/cm3,
4100 reflections measured (8.174° ≤ 2Θ ≤ 134.138°),
2032 unique (*R*int = 0.0304, Rsigma = 0.0405) which were used in all calculations.
The final *R*1 was 0.0366
(I > 2σ(I)) and *wR*2 was 0.0924 (all data).

Refinement model description

Number of restraints - 0,
number of constraints - unknown.

Details:

```
1. Fixed Uiso
```

This report has been created with Olex2, compiled on
2018.04.03 svn.r3497 for OlexSys. Please
let us know
if there are any errors or if you would like to have additional features.
