# Supplementary material for: Investigation of 8-Aza-7-Deaza Purine Nucleoside Derivatives
Source: Molecules. 2019 Mar 11;24(5):983. doi: 10.3390/molecules24050983 (PMC6429420; doi:10.3390/molecules24050983)

checkCIF/PLATON report

Structure factors have been supplied for datablock(s) 201804211

THIS REPORT IS FOR GUIDANCE ONLY. IF USED AS PART OF A REVIEW PROCEDURE FOR PUBLICATION, IT SHOULD NOT REPLACE THE EXPERTISE OF AN EXPERIENCED CRYSTALLOGRAPHIC REFEREE.

No syntax errors found.   CIF dictionary   Interpreting this report

Datablock: 201804211

```
Bond precision:  C-C = 0.0046 A          Wavelength=1.54184

Cell:             a=4.7669(2)     b=11.0409(5)     c=21.6334(9)
                  alpha=90        beta=90          gamma=90
Temperature:      293 K

                Calculated                 Reported
Volume          1138.59(8)                 1138.59(8)
Space group     P 21 21 21                 P 21 21 21
Hall group      P 2ac 2ab                  P 2ac 2ab
Moiety formula  C10 H13 N5 O5              C10 H13 N5 O5
Sum formula     C10 H13 N5 O5              C10 H13 N5 O5
Mr              283.25                     283.25
Dx,g cm-3       1.652                      1.652
Z               4                          4
Mu (mm-1)       1.157                      1.157
F000            592.0                      592.0
F000'           594.18
h,k,lmax        5,13,25                    5,13,25
Nref            2038[ 1228]                2032
Tmin,Tmax       0.858,0.891                0.977,1.000
Tmin'           0.841

Correction method= # Reported T Limits: Tmin=0.977 Tmax=1.000
AbsCorr = MULTI-SCAN

Data completeness= 1.65/1.00      Theta(max)= 67.069

R(reflections)= 0.0366( 1873)     wR2(reflections)= 0.0924( 2032)

S = 1.036                 Npar= 184
```

The following ALERTS were generated. Each ALERT has the format
test-name_ALERT_alert-type_alert-level.
Click on the hyperlinks for more details of the test.

Alert level C

```
PLAT089_ALERT_3_C Poor Data / Parameter Ratio (Zmax < 18) ........       6.67 Note
PLAT340_ALERT_3_C Low Bond Precision on  C-C Bonds ...............    0.00457 Ang.
PLAT911_ALERT_3_C Missing FCF Refl Between Thmin & STh/L=    0.597          2 Report
```

Alert level G

```
PLAT007_ALERT_5_G Number of Unrefined Donor-H Atoms ..............          6 Report
PLAT012_ALERT_1_G No       _shelx_res_checksum Found in CIF ......     Please Check
PLAT199_ALERT_1_G Reported _cell_measurement_temperature ..... (K)        293 Check
PLAT200_ALERT_1_G Reported   _diffrn_ambient_temperature ..... (K)        293 Check
PLAT398_ALERT_2_G Deviating  C-O-C    Angle From 120 for O1             108.3 Degree
PLAT720_ALERT_4_G Number of Unusual/Non-Standard Labels ..........          2 Note
PLAT791_ALERT_4_G Model has Chirality at C1'         (Chiral SPGR)          R Verify
PLAT791_ALERT_4_G Model has Chirality at C2'         (Chiral SPGR)          R Verify
PLAT791_ALERT_4_G Model has Chirality at C3'         (Chiral SPGR)          S Verify
PLAT791_ALERT_4_G Model has Chirality at C4'         (Chiral SPGR)          R Verify
PLAT909_ALERT_3_G Percentage of I>2sig(I) Data at Theta(Max) Still        87% Note
PLAT978_ALERT_2_G Number C-C Bonds with Positive Residual Density.          3 Info
```

---

0 **ALERT level A** = Most likely a serious problem - resolve or explain
0 **ALERT level B** = A potentially serious problem, consider carefully
3 **ALERT level C** = Check. Ensure it is not caused by an omission or oversight
12 **ALERT level G** = General information/check it is not something unexpected

3 ALERT type 1 CIF construction/syntax error, inconsistent or missing data
2 ALERT type 2 Indicator that the structure model may be wrong or deficient
4 ALERT type 3 Indicator that the structure quality may be low
5 ALERT type 4 Improvement, methodology, query or suggestion
1 ALERT type 5 Informative message, check

It is advisable to attempt to resolve as many as possible of the alerts in all categories. Often the minor alerts point to easily fixed oversights, errors and omissions in your CIF or refinement strategy, so attention to these fine details can be worthwhile. In order to resolve some of the more serious problems it may be necessary to carry out additional measurements or structure refinements. However, the purpose of your study may justify the reported deviations and the more serious of these should normally be commented upon in the discussion or experimental section of a paper or in the "special_details" fields of the CIF. checkCIF was carefully designed to identify outliers and unusual parameters, but every test has its limitations and alerts that are not important in a particular case may appear. Conversely, the absence of alerts does not guarantee there are no aspects of the results needing attention. It is up to the individual to critically assess their own results and, if necessary, seek expert advice.

Publication of your CIF in IUCr journals

A basic structural check has been run on your CIF. These basic checks will be run on all CIFs submitted for publication in IUCr journals (*Acta Crystallographica*, *Journal of Applied Crystallography*, *Journal of Synchrotron Radiation*); however, if you intend to submit to *Acta Crystallographica Section C* or *E* or *IUCrData*, you should make sure that full publication checks are run on the final version of your CIF prior to submission.

Publication of your CIF in other journals

Please refer to the *Notes for Authors* of the relevant journal for any special instructions relating to CIF submission.

PLATON version of 30/01/2018; check.def file version of 30/01/2018

Datablock 201804211 - ellipsoid plot

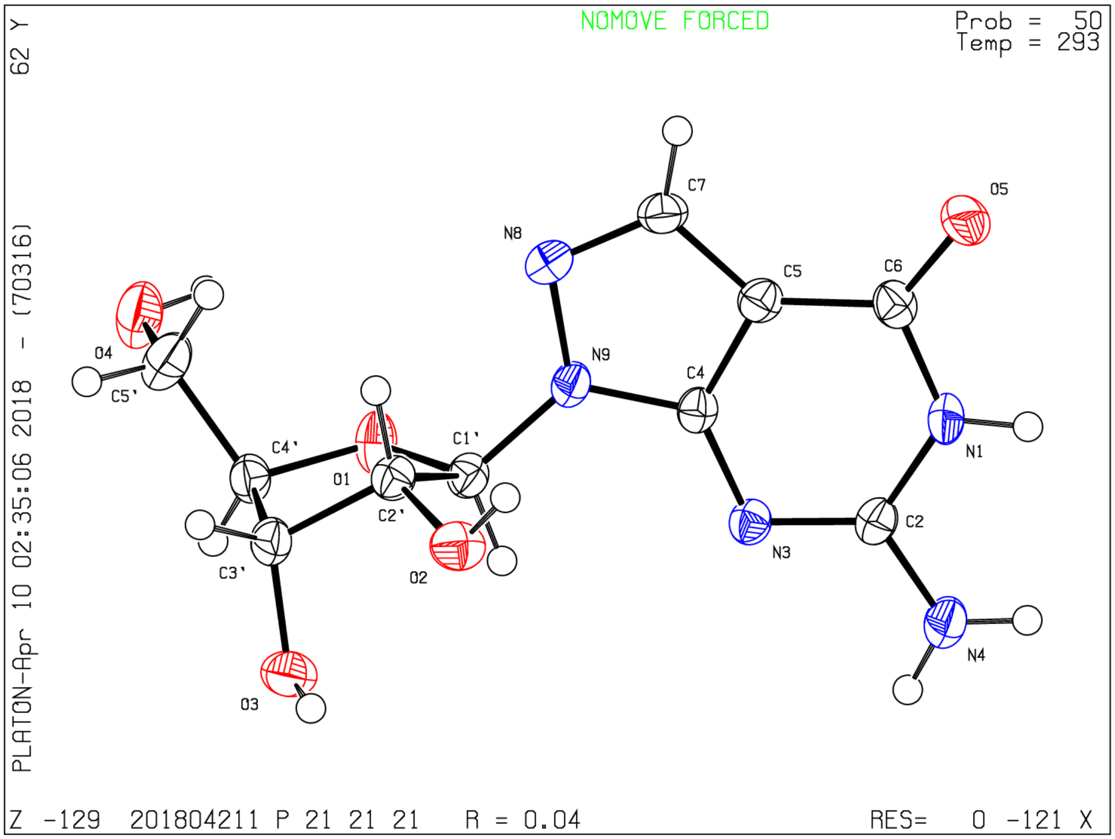

Supplement: Supplementary file 1 [file molecules-24-00983-s001.zip › molecules-460200 proof supple/supplementory materials Dr. An/cpd 7-Crystallographic information/checkcif-7.pdf]
