# Supplementary material for: Investigation of 8-Aza-7-Deaza Purine Nucleoside Derivatives
Source: Molecules. 2019 Mar 11;24(5):983. doi: 10.3390/molecules24050983 (PMC6429420; doi:10.3390/molecules24050983)

checkCIF/PLATON report

Structure factors have been supplied for datablock(s) 201804210

THIS REPORT IS FOR GUIDANCE ONLY. IF USED AS PART OF A REVIEW PROCEDURE FOR PUBLICATION, IT SHOULD NOT REPLACE THE EXPERTISE OF AN EXPERIENCED CRYSTALLOGRAPHIC REFEREE.

No syntax errors found. CIF dictionary Interpreting this report

Datablock: 201804210

```
Bond precision:  C-C = 0.0093 A          Wavelength=1.54184

Cell:           a=5.08845(13)    b=12.7514(2)        c=11.3200(2)
                alpha=90         beta=91.4146(19)    gamma=90
Temperature:    293 K

                Calculated                   Reported
Volume          734.27(3)                    734.28(3)
Space group     P 21                         P 1 21 1
Hall group      P 2yb                        P 2yb
Moiety formula  C10 H13 I N6 O4, H2 O        C10 H13 I N6 O4, H2 O
Sum formula     C10 H15 I N6 O5              C10 H15 I N6 O5
Mr              426.18                       426.18
Dx,g cm-3       1.928                        1.928
Z               2                            2
Mu (mm-1)       17.478                       17.478
F000            420.0                        420.0
F000'           420.74
h,k,lmax        6,15,13                      6,15,13
Nref            2619[ 1373]                  2616
Tmin,Tmax       0.138,0.146                  0.253,1.000
Tmin'           0.038

Correction method= # Reported T Limits: Tmin=0.253 Tmax=1.000
AbsCorr = MULTI-SCAN

Data completeness= 1.91/1.00      Theta(max)= 67.047

R(reflections)= 0.0286( 2551)     wR2(reflections)= 0.0711( 2616)

S = 1.062                  Npar= 213
```

The following ALERTS were generated. Each ALERT has the format
test-name_ALERT_alert-type_alert-level.
Click on the hyperlinks for more details of the test.

Alert level C

```
PLAT090_ALERT_3_C Poor Data / Parameter Ratio (Zmax > 18) ........       6.45 Note
PLAT342_ALERT_3_C Low Bond Precision on  C-C Bonds ...............    0.00929 Ang.
PLAT420_ALERT_2_C D-H Without Acceptor       N5       --H5B         Please Check
PLAT480_ALERT_4_C Long H...A H-Bond Reported H4B      ..O5             2.64 Ang.
PLAT480_ALERT_4_C Long H...A H-Bond Reported H5C      ..O3             2.69 Ang.
PLAT978_ALERT_2_C Number C-C Bonds with Positive Residual Density.        0 Info
```

Alert level G

```
PLAT002_ALERT_2_G Number of Distance or Angle Restraints on AtSite        3 Note
PLAT007_ALERT_5_G Number of Unrefined Donor-H Atoms ..............        7 Report
PLAT012_ALERT_1_G No      _shelx_res_checksum Found in CIF ......   Please Check
PLAT172_ALERT_4_G The CIF-Embedded .res File Contains DFIX Records        2 Report
PLAT199_ALERT_1_G Reported _cell_measurement_temperature ..... (K)      293 Check
PLAT200_ALERT_1_G Reported   _diffrn_ambient_temperature ..... (K)      293 Check
PLAT398_ALERT_2_G Deviating  C-O-C    Angle From 120 for O1           105.4 Degree
PLAT720_ALERT_4_G Number of Unusual/Non-Standard Labels ..........        2 Note
PLAT791_ALERT_4_G Model has Chirality at C1’         (Chiral SPGR)        R Verify
PLAT791_ALERT_4_G Model has Chirality at C2’         (Chiral SPGR)        R Verify
PLAT791_ALERT_4_G Model has Chirality at C3’         (Chiral SPGR)        S Verify
PLAT791_ALERT_4_G Model has Chirality at C4’         (Chiral SPGR)        R Verify
PLAT860_ALERT_3_G Number of Least-Squares Restraints .............        3 Note
PLAT909_ALERT_3_G Percentage of I>2sig(I) Data at Theta(Max) Still       96% Note
```

---

0 **ALERT level A** = Most likely a serious problem - resolve or explain
0 **ALERT level B** = A potentially serious problem, consider carefully
6 **ALERT level C** = Check. Ensure it is not caused by an omission or oversight
14 **ALERT level G** = General information/check it is not something unexpected

3 ALERT type 1 CIF construction/syntax error, inconsistent or missing data
4 ALERT type 2 Indicator that the structure model may be wrong or deficient
4 ALERT type 3 Indicator that the structure quality may be low
8 ALERT type 4 Improvement, methodology, query or suggestion
1 ALERT type 5 Informative message, check

It is advisable to attempt to resolve as many as possible of the alerts in all categories. Often the minor alerts point to easily fixed oversights, errors and omissions in your CIF or refinement strategy, so attention to these fine details can be worthwhile. In order to resolve some of the more serious problems it may be necessary to carry out additional measurements or structure refinements. However, the purpose of your study may justify the reported deviations and the more serious of these should normally be commented upon in the discussion or experimental section of a paper or in the "special_details" fields of the CIF. checkCIF was carefully designed to identify outliers and unusual parameters, but every test has its limitations and alerts that are not important in a particular case may appear. Conversely, the absence of alerts does not guarantee there are no aspects of the results needing attention. It is up to the individual to critically assess their own results and, if necessary, seek expert advice.

Publication of your CIF in IUCr journals

A basic structural check has been run on your CIF. These basic checks will be run on all CIFs submitted for publication in IUCr journals (*Acta Crystallographica*, *Journal of Applied Crystallography*, *Journal of Synchrotron Radiation*); however, if you intend to submit to *Acta Crystallographica Section C* or *E* or *IUCrData*, you should make sure that full publication checks are run on the final version of your CIF prior to submission.

Publication of your CIF in other journals

Please refer to the *Notes for Authors* of the relevant journal for any special instructions relating to CIF submission.

PLATON version of 30/01/2018; check.def file version of 30/01/2018

Datablock 201804210 - ellipsoid plot

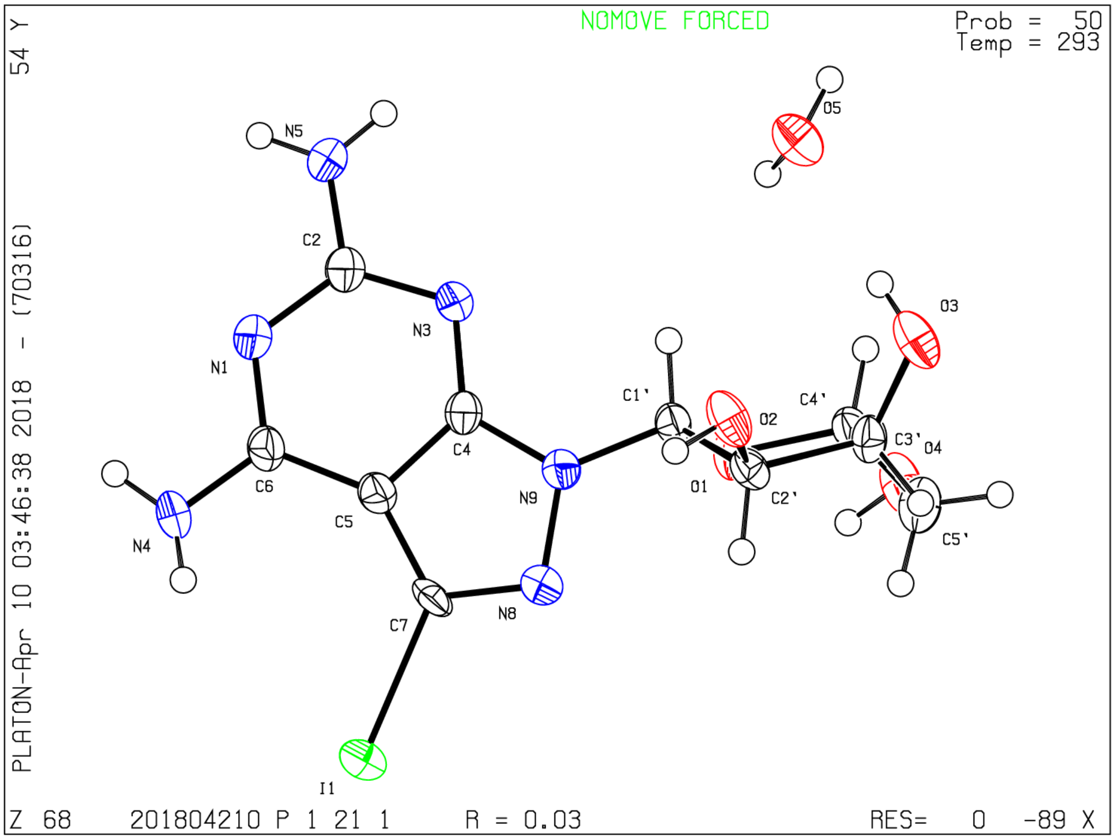

Supplement: Supplementary file 1 [file molecules-24-00983-s001.zip › molecules-460200 proof supple/supplementory materials Dr. An/cpd 8-Crystallographic information/checkcif-8.pdf]
