# Supplementary material for: Investigation of 8-Aza-7-Deaza Purine Nucleoside Derivatives
Source: Molecules. 2019 Mar 11;24(5):983. doi: 10.3390/molecules24050983 (PMC6429420; doi:10.3390/molecules24050983)
Supplement: Supplementary file 1 [file molecules-24-00983-s001.zip › molecules-460200 proof supple/supplementory materials Dr. An/Supplementary Materials spectra and data.pdf]

Supplementary Materials

# Investigation of 8-aza-7-deaza purine nucleoside derivatives

**Hang Ren [1,2], Haoyun An [2,*] and Jingchao Tao [1,*]**

## Table of contents

$^{1}$H NMR spectra of product **1** and **2**……………………………..…………S2

$^{1}$H NMR spectra of product **3** and **5**……………………………...………S3

$^{1}$H NMR spectra of product **7** and **8**……………………………...………S4

$^{1}$H NMR spectra of product **10** and **12**…………………………………………S5

$^{1}$H NMR spectra of product **13** and **14**……………………….…..………S6

$^{1}$H NMR spectra of product **15** and **16**……………………..……………..S7

$^{1}$H NMR spectra of product **17** and **18**………………………..……….....S8

$^{1}$H NMR spectra of product **19** and **22**……………………….……….......S9

$^{1}$H NMR spectra of product **23** and **27**……………………….……………..S10

$^{13}$C NMR spectra of product **7** and **10**……………………………………S11

HRMS spectra of product **2** and **6**……………………………..……......S12

HRMS spectra of product **12** and **13**………………………………….……..S13

HRMS spectra of product **14** and **15**……………………………....…...S14

HRMS spectra of product **16**……………………………………….……..…S15

Crystal data and structure refinement for compound **7**.…………………..S16

Crystal data and structure refinement for compound **8**…………………..S17

**Product 1:** $^1$H NMR (400 MHz, DMSO-$d_6$)

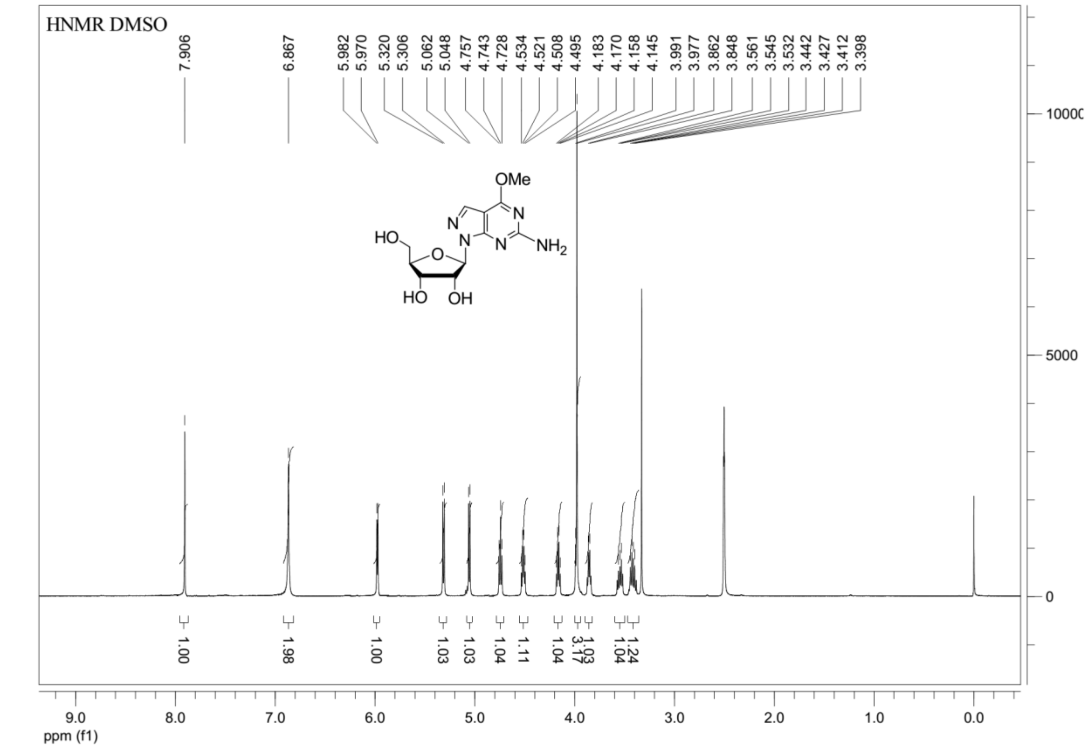


**Product 2:** $^1$H NMR (400 MHz, DMSO-$d_6$)

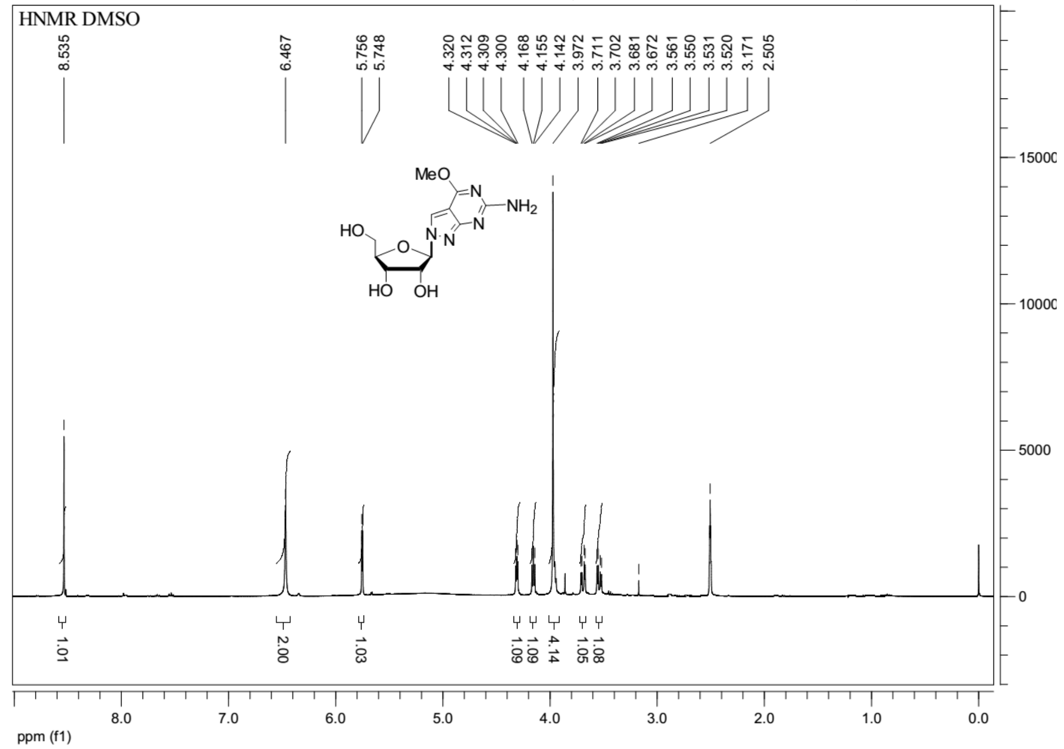

**Product 3:** $^{1}$H NMR (400 MHz, DMSO-*d*$_6$)

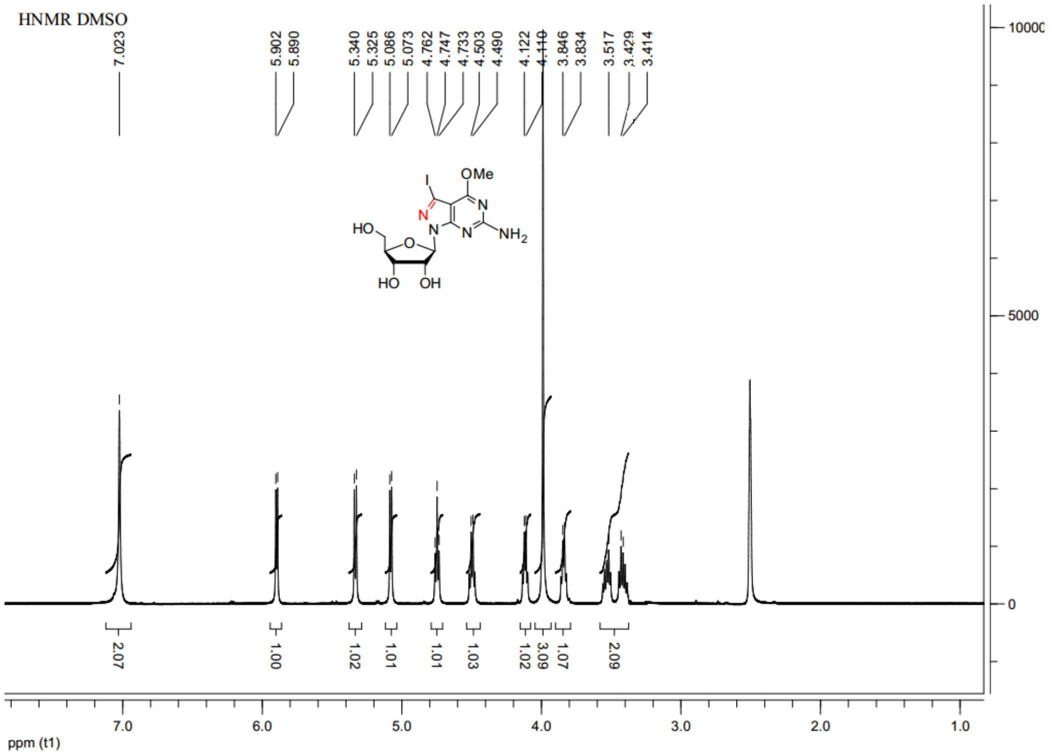


**Product 5:** $^{1}$H NMR (400 MHz, DMSO-*d*$_6$)

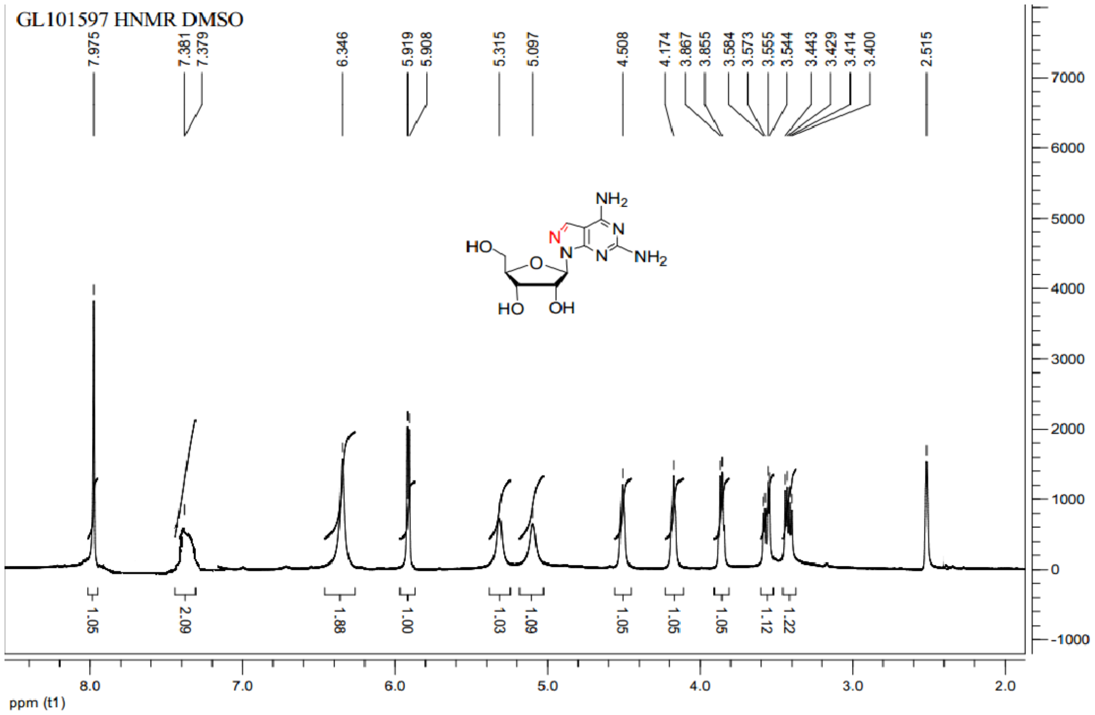

**Product 7:** $^{1}$H NMR (400 MHz, DMSO-$d_6$)

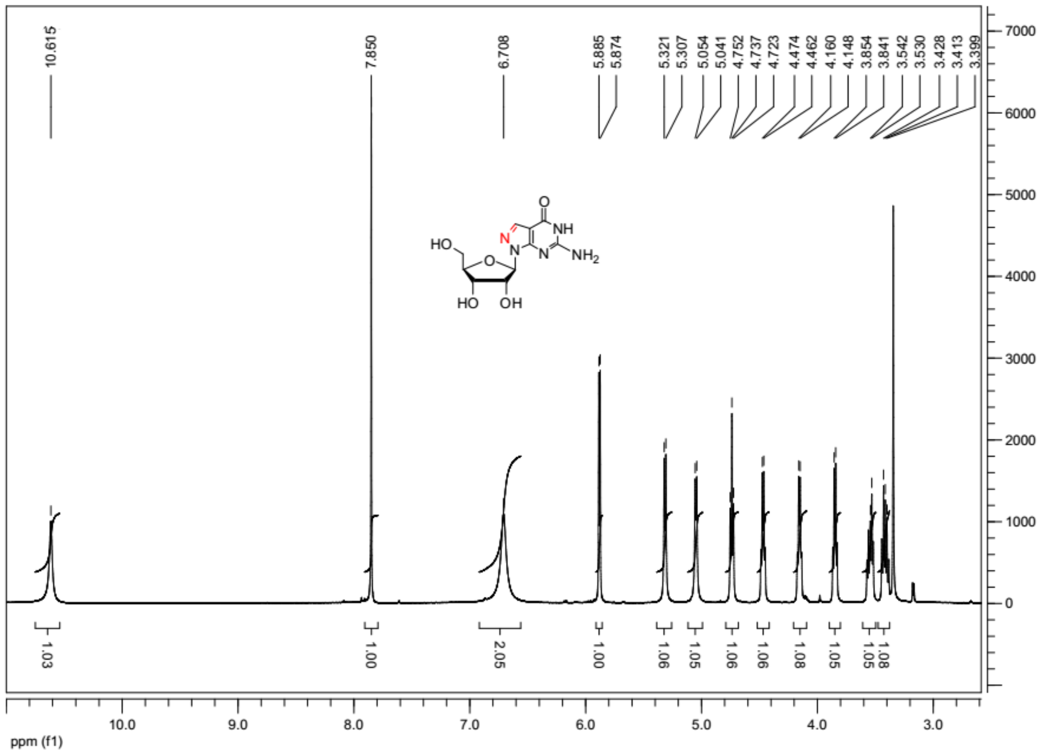


**Product 8:** $^{1}$H NMR (400 MHz, DMSO-$d_6$)

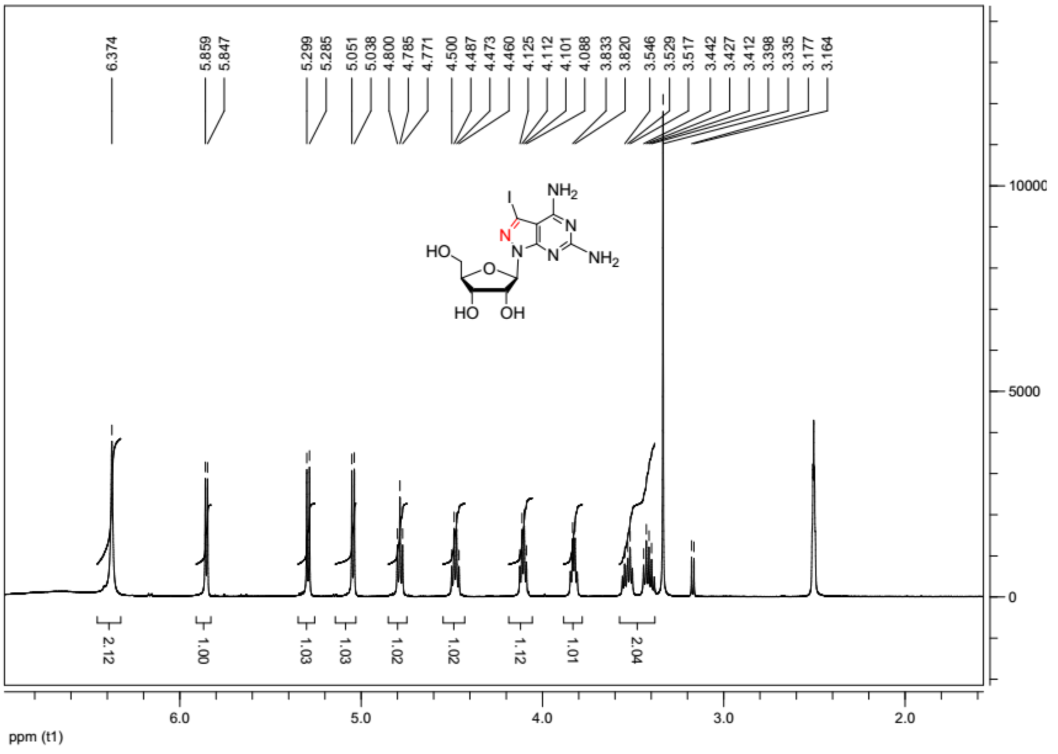

**Product 10:** $^{1}$H NMR (400 MHz, DMSO-$d_6$)

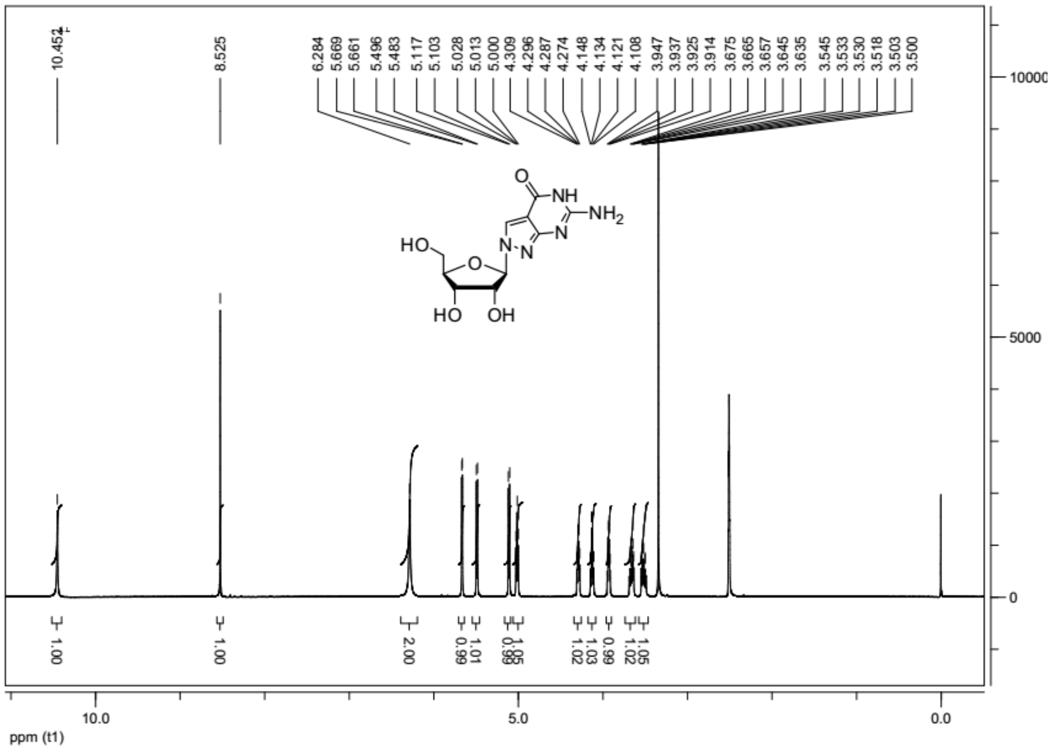


**Product 12:** $^{1}$H NMR (400 MHz, DMSO-$d_6$)

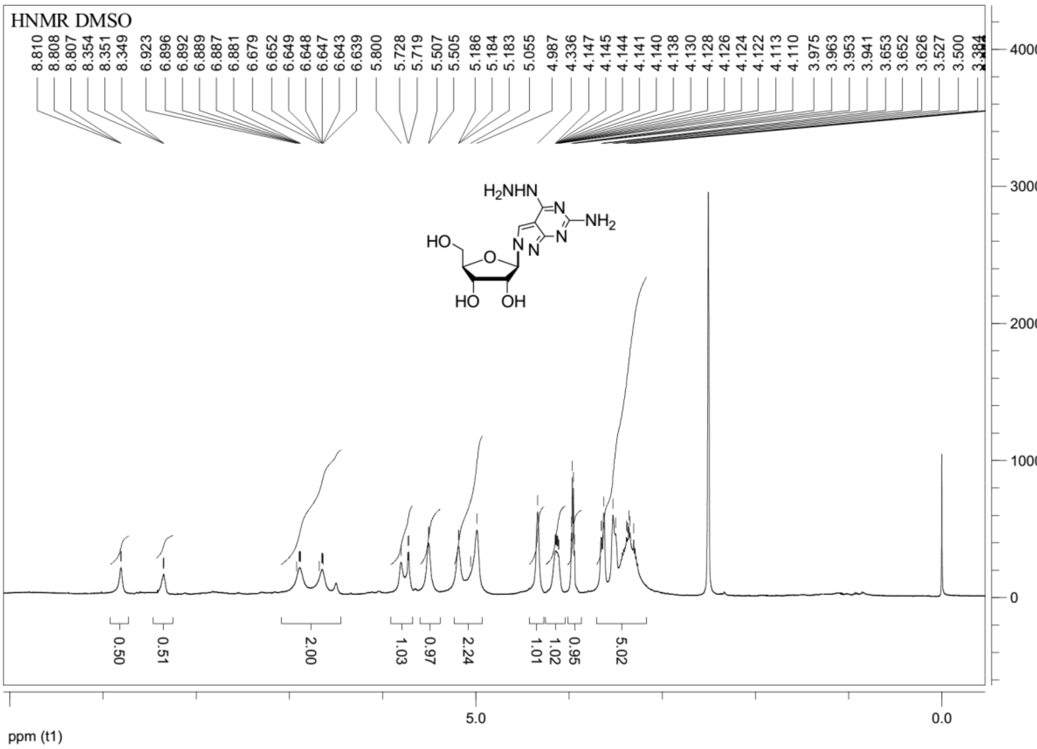

**Product 13:** $^1$H NMR (400 MHz, DMSO-$d_6$)

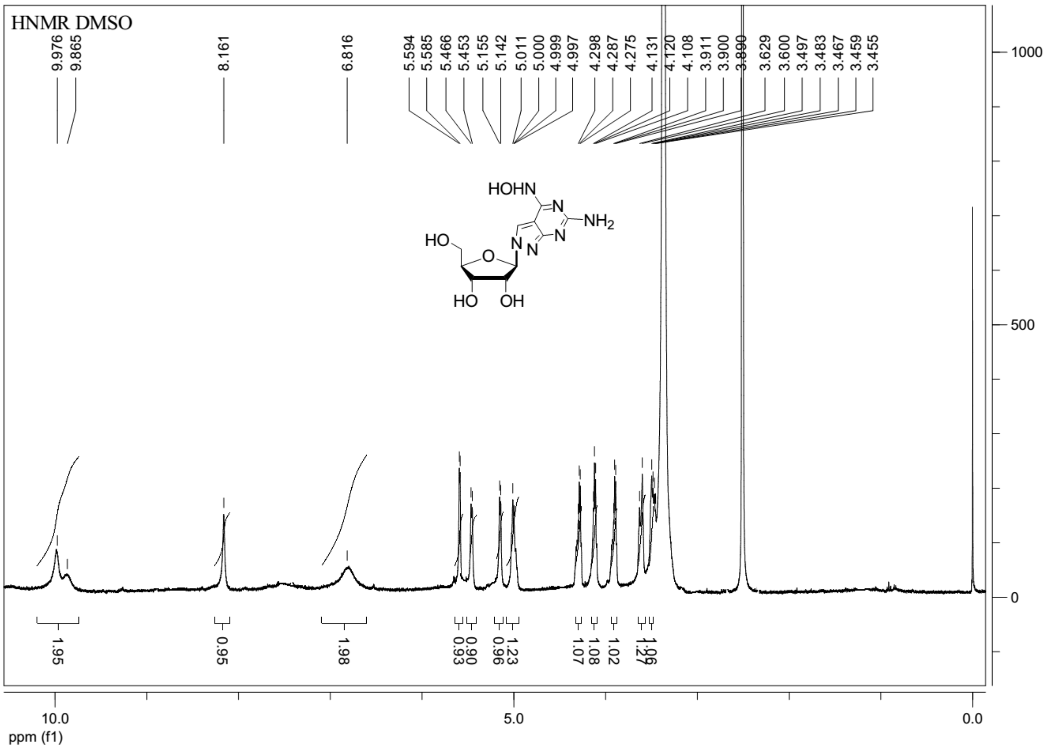

**Product 14:** $^1$H NMR (400 MHz, DMSO-$d_6$)

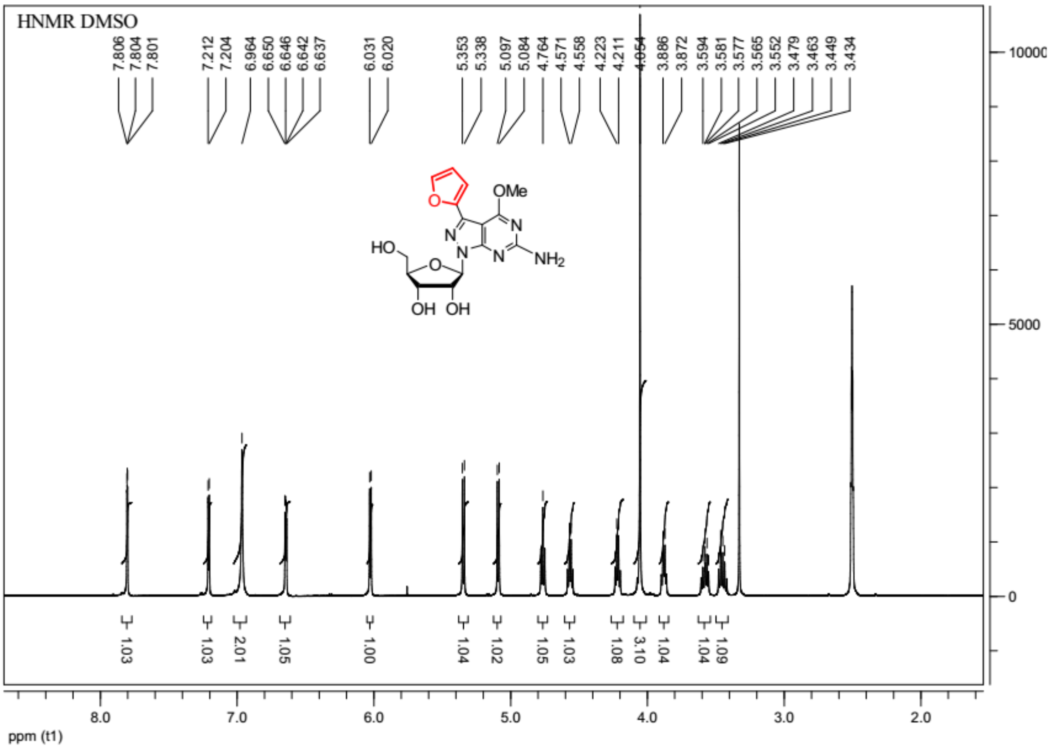

**Product 15:** $^{1}$H NMR (400 MHz, DMSO-$d_6$)

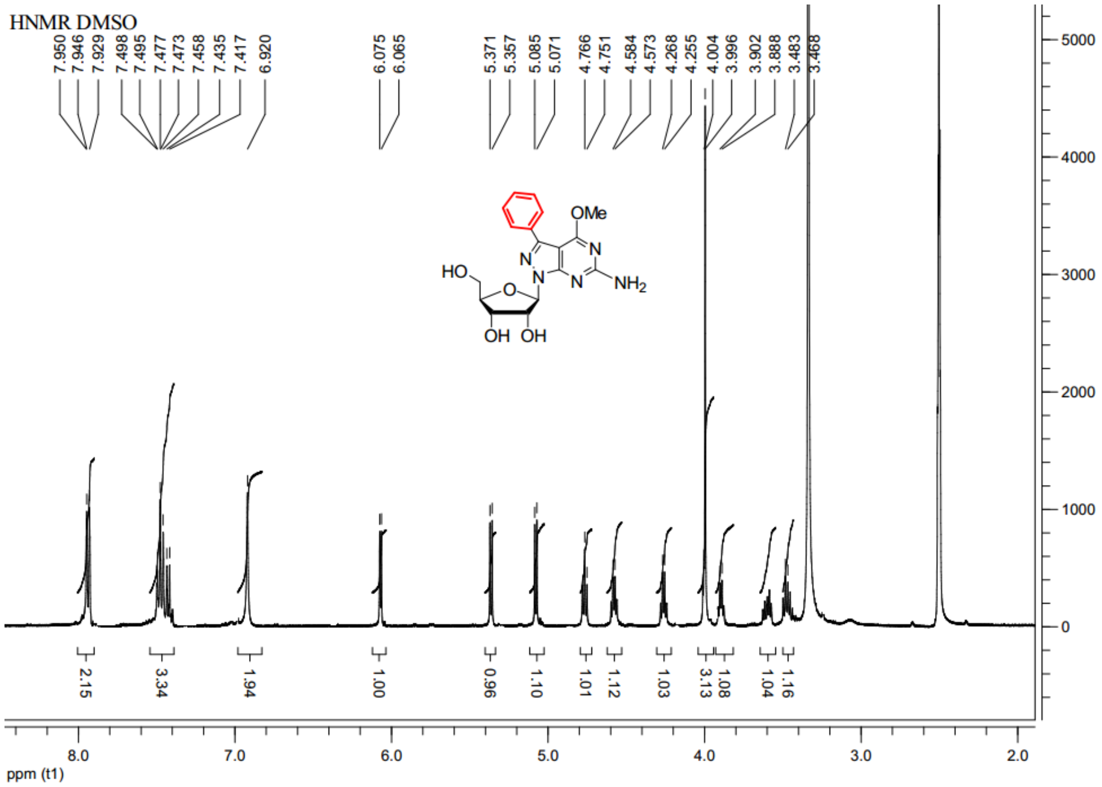


**Product 16:** $^{1}$H NMR (400 MHz, DMSO-$d_6$)

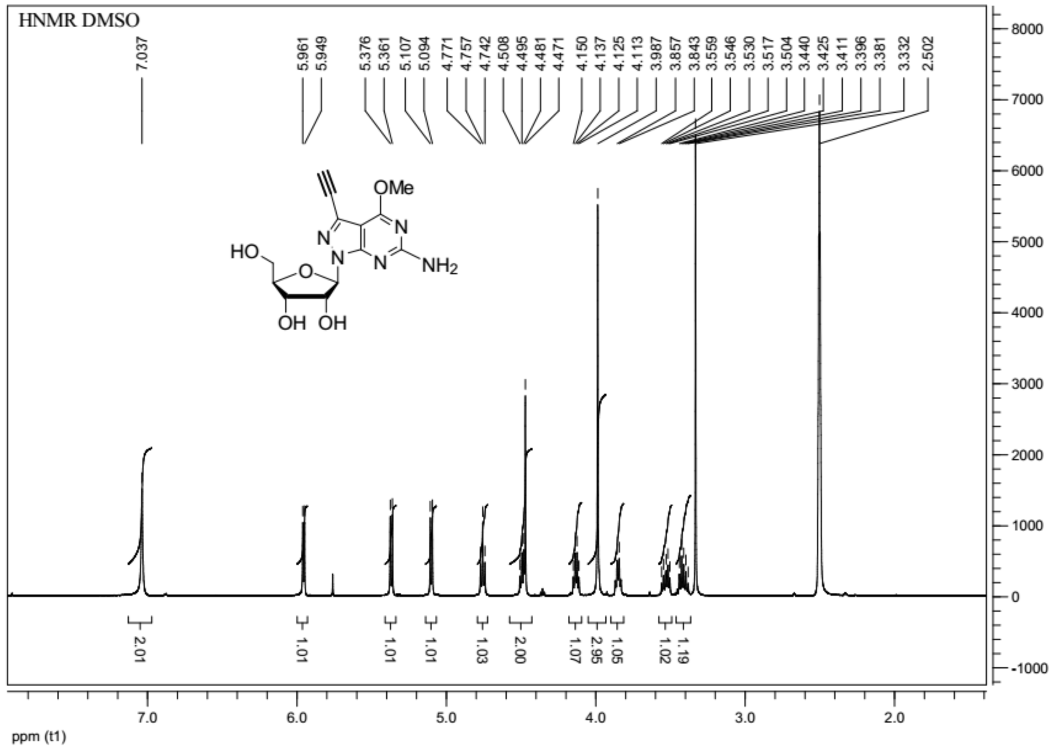

**Product 17:** ¹H NMR (400 MHz, DMSO-$d_6$)

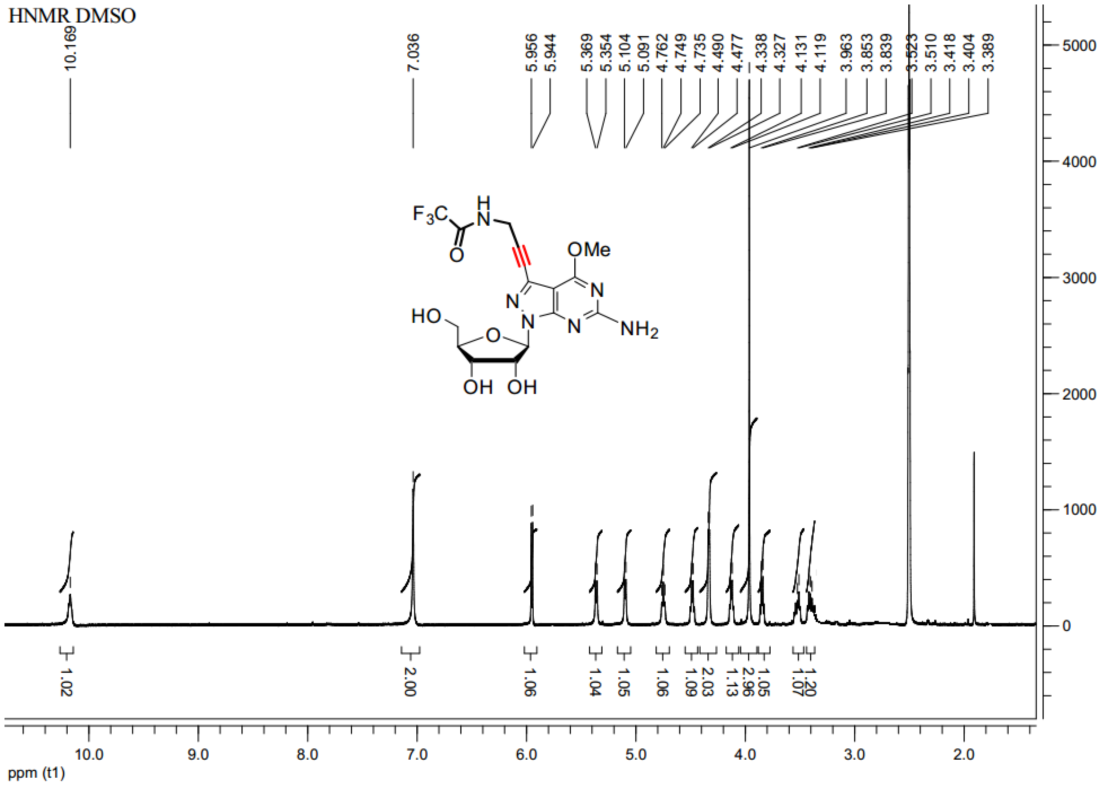

**Product 18:** ¹H NMR (400 MHz, DMSO-$d_6$)

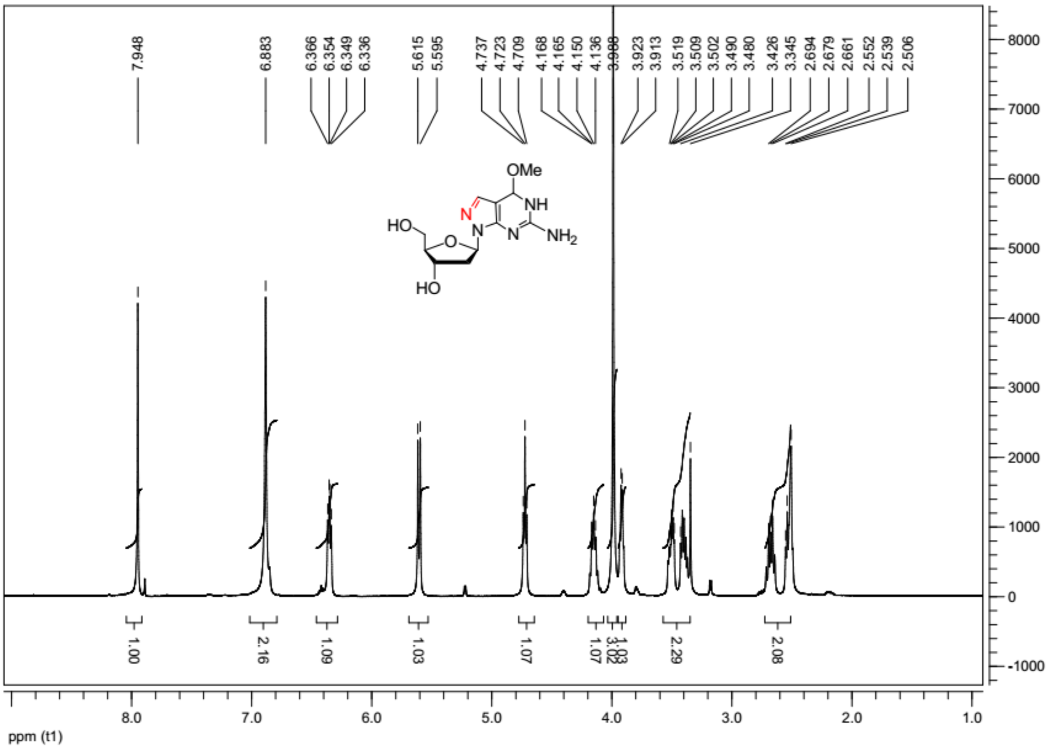

**Product 19:** $^1$H NMR (400 MHz, DMSO-$d_6$)

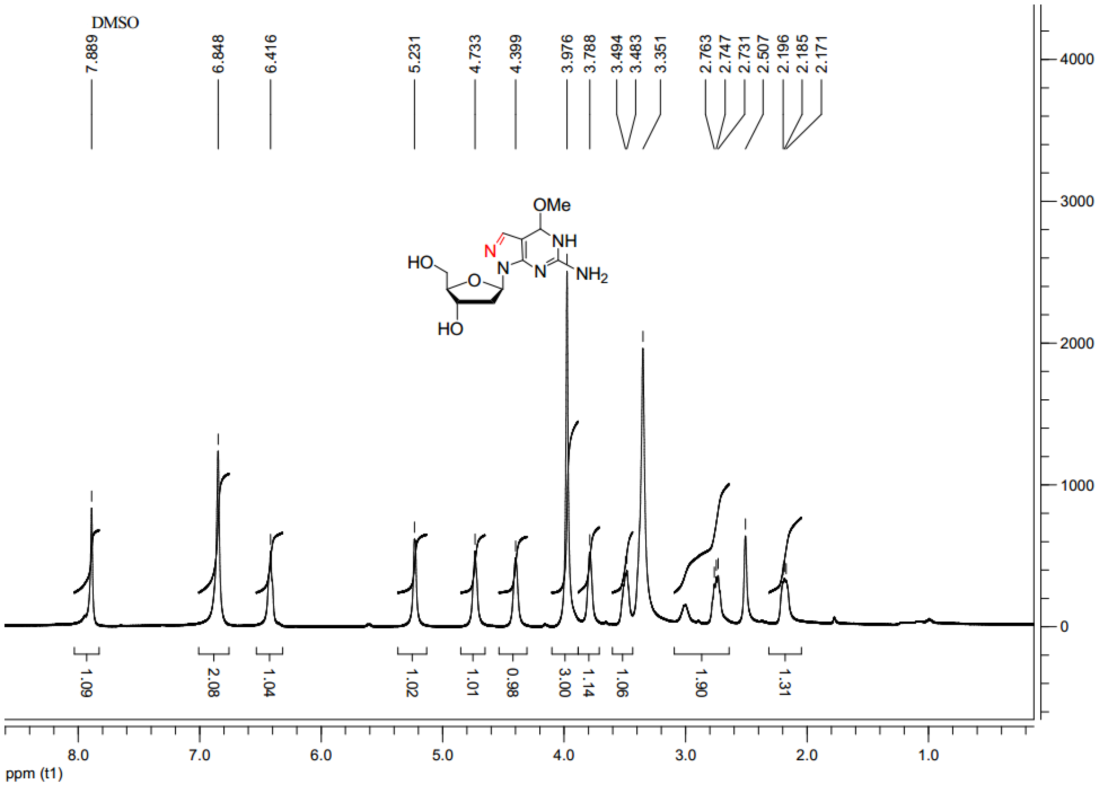

**Product 22:** $^1$H NMR (400 MHz, DMSO-$d_6$)

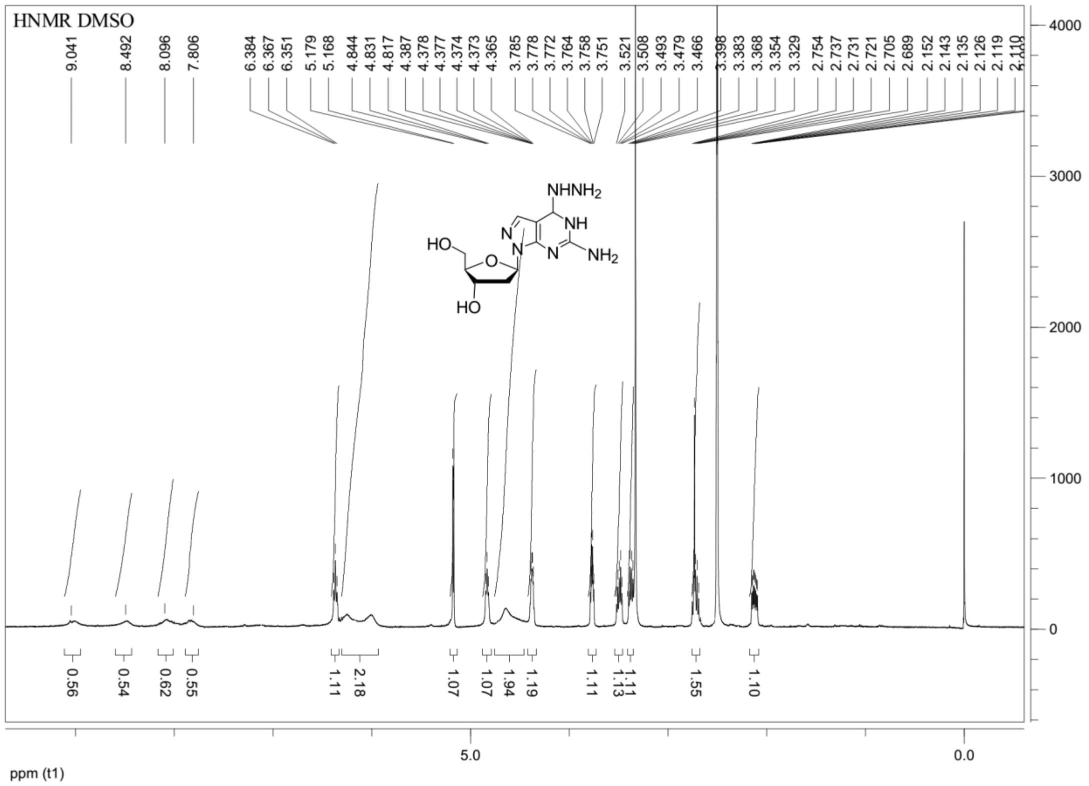

**Product 23:** $^1$H NMR (400 MHz, DMSO-$d_6$)

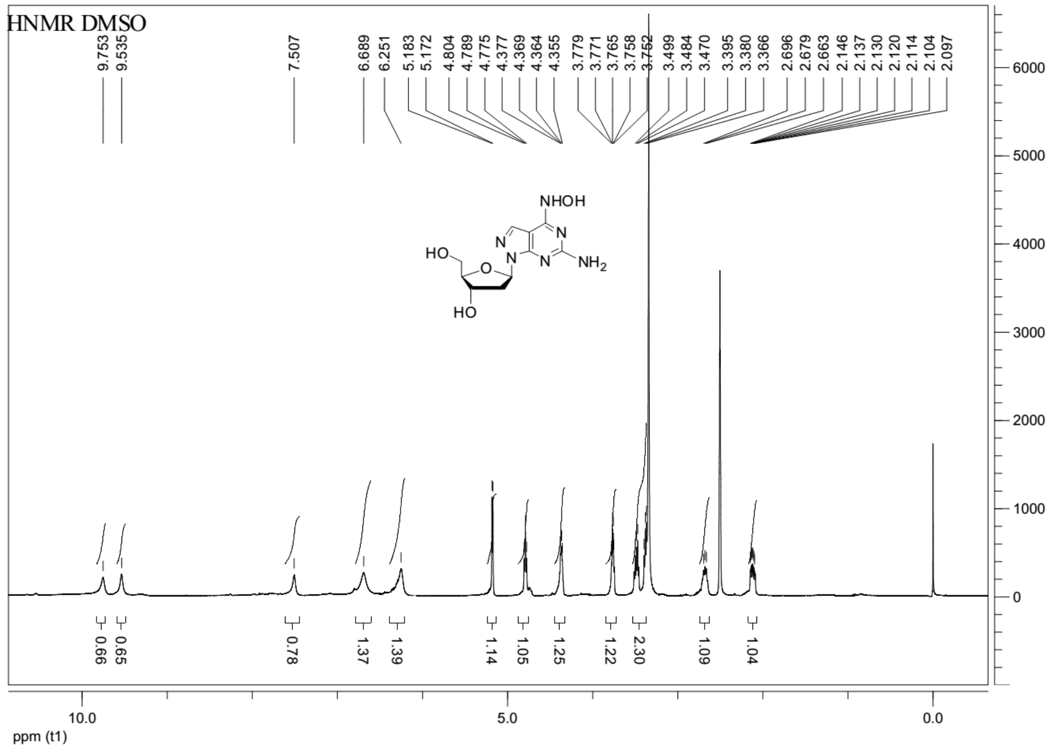

**Product 27:** $^1$H NMR (400 MHz, DMSO-$d_6$)

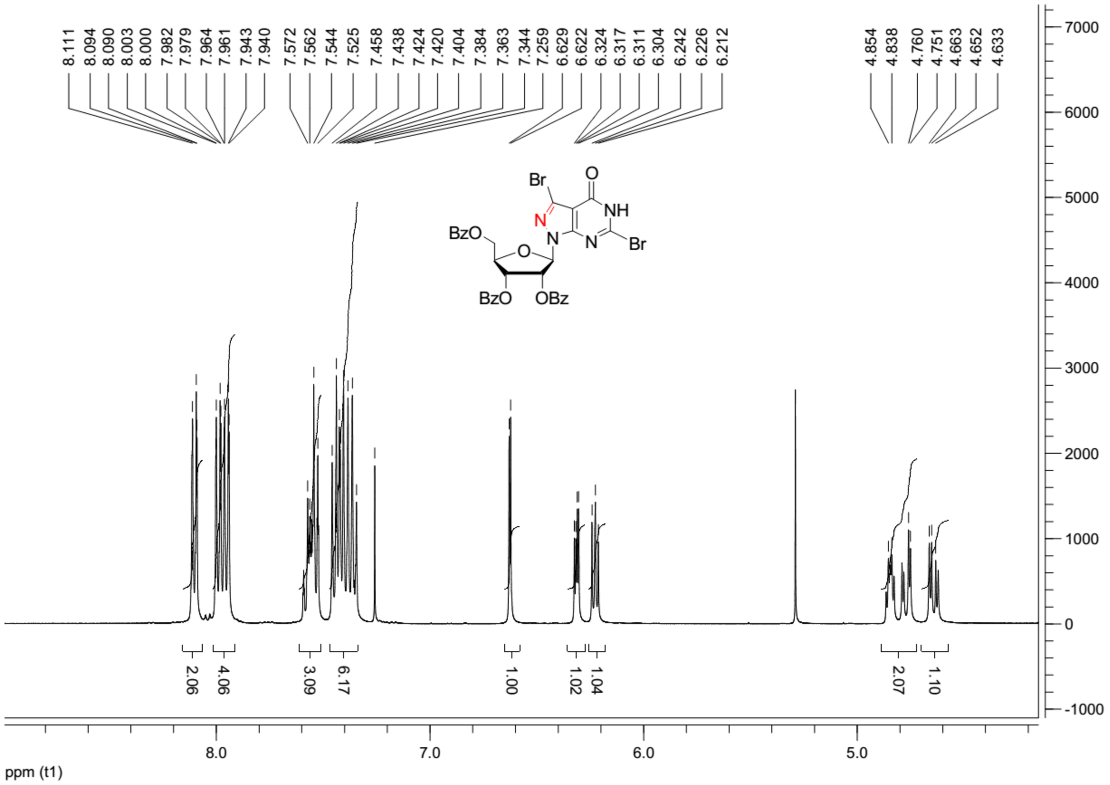

**Product 7:** $^{13}$C NMR (400 MHz, DMSO-$d_6$)

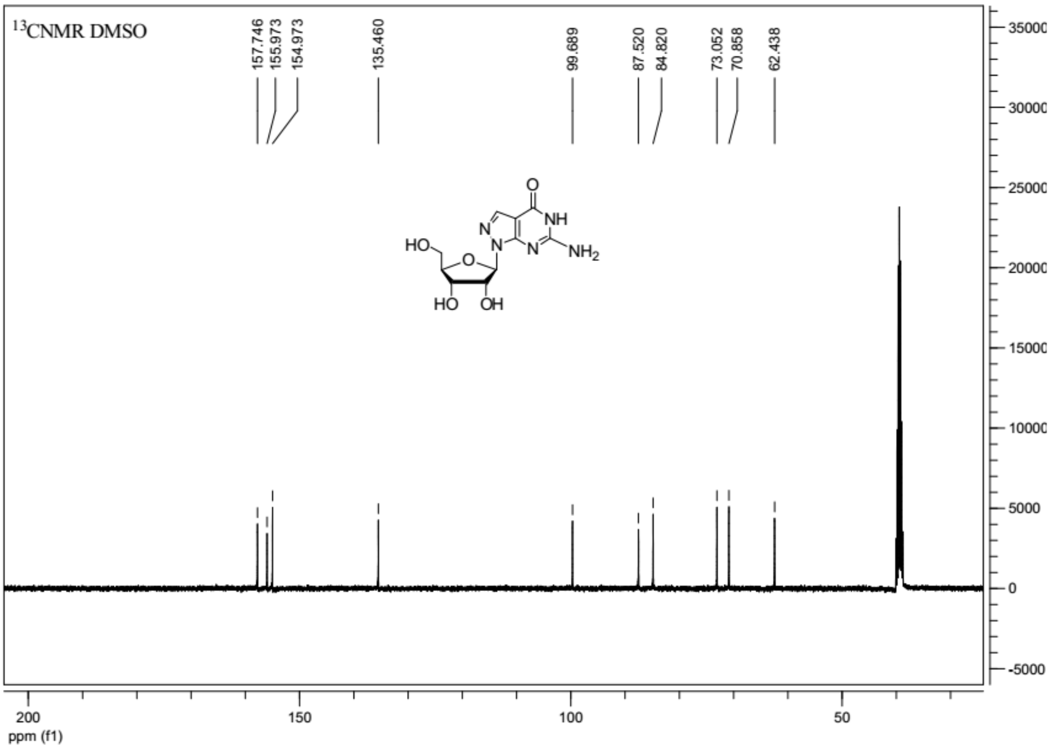


**Product 10:** $^{13}$C NMR (400 MHz, DMSO-$d_6$)

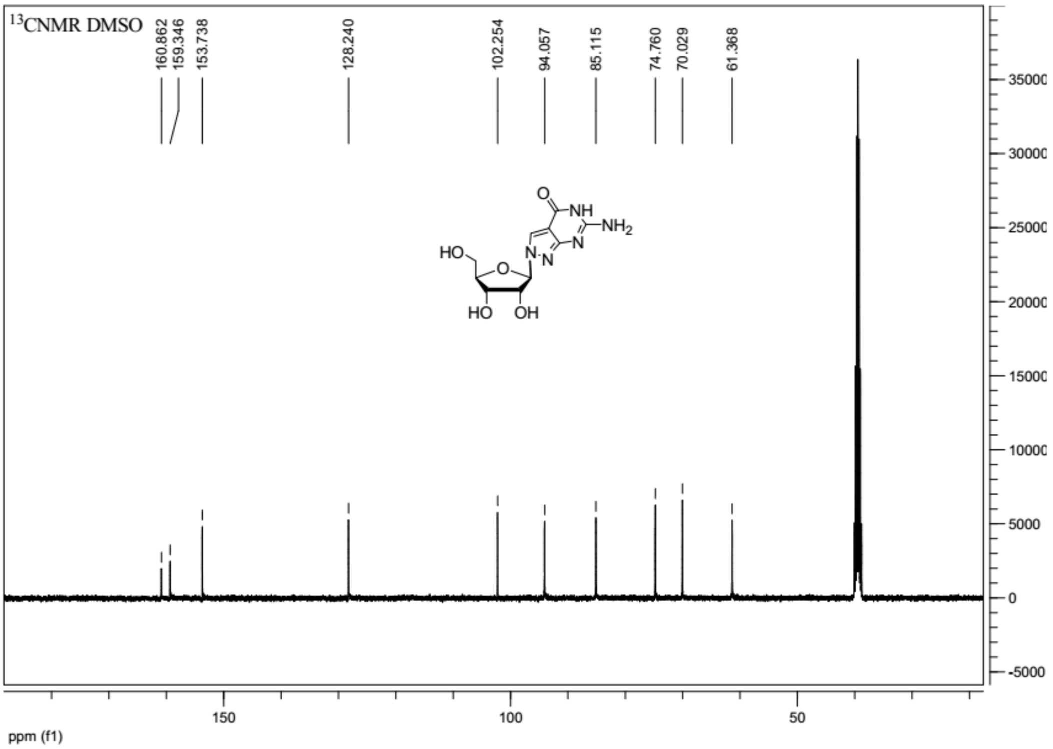

## Product 2: HRMS spectra

2

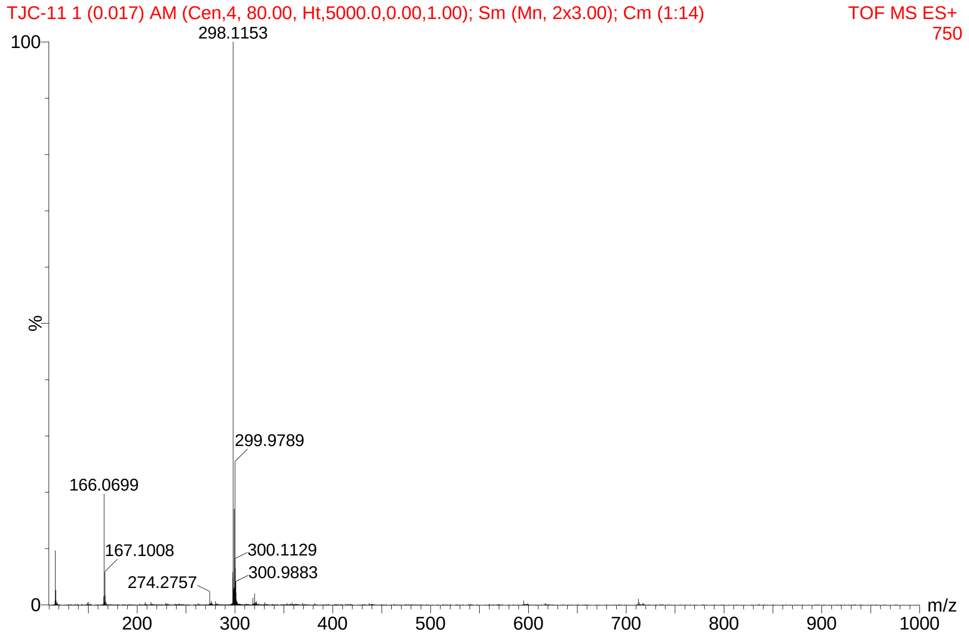


## Product 6: HRMS spectra

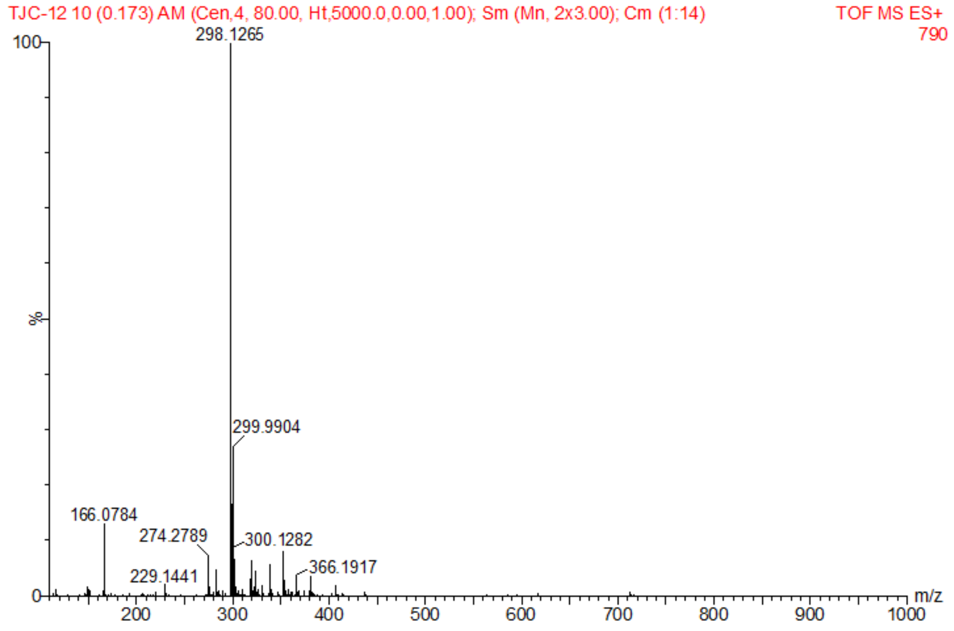

## Product 12: HRMS spectra

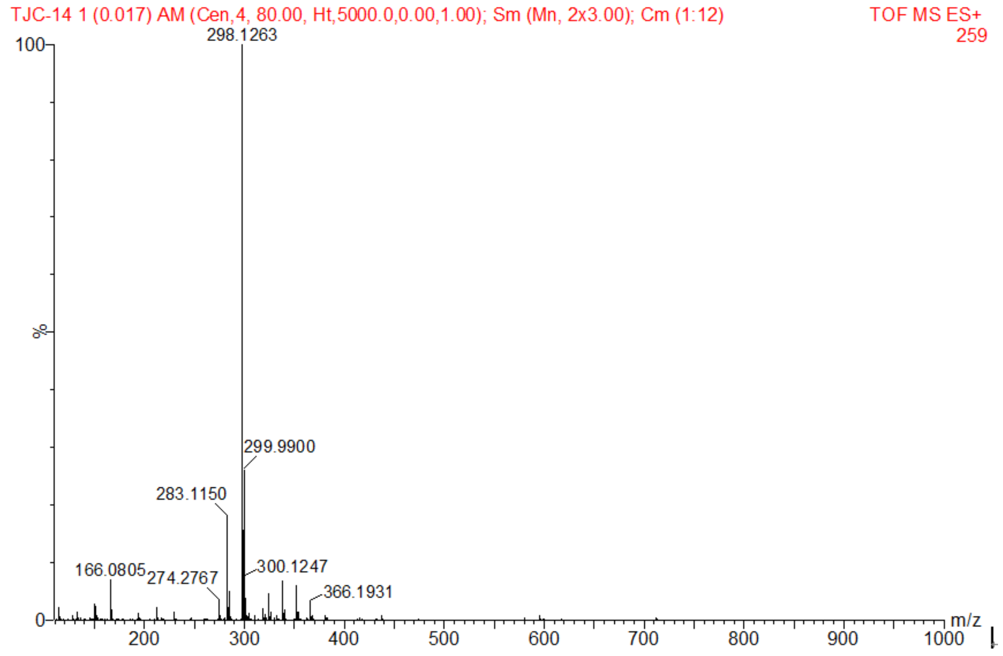


## Product 13: HRMS spectra

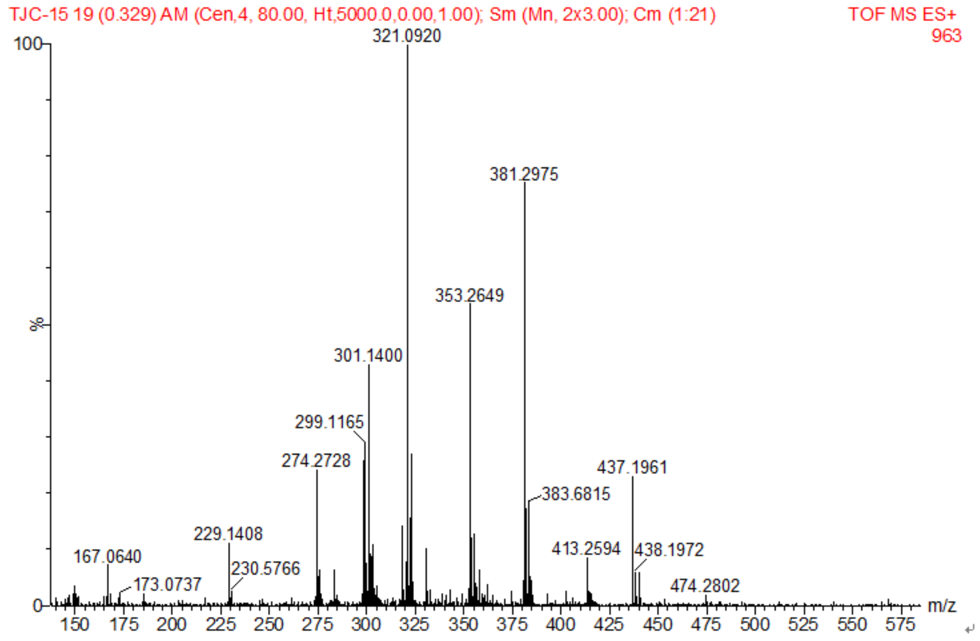

## Product 14: HRMS spectra

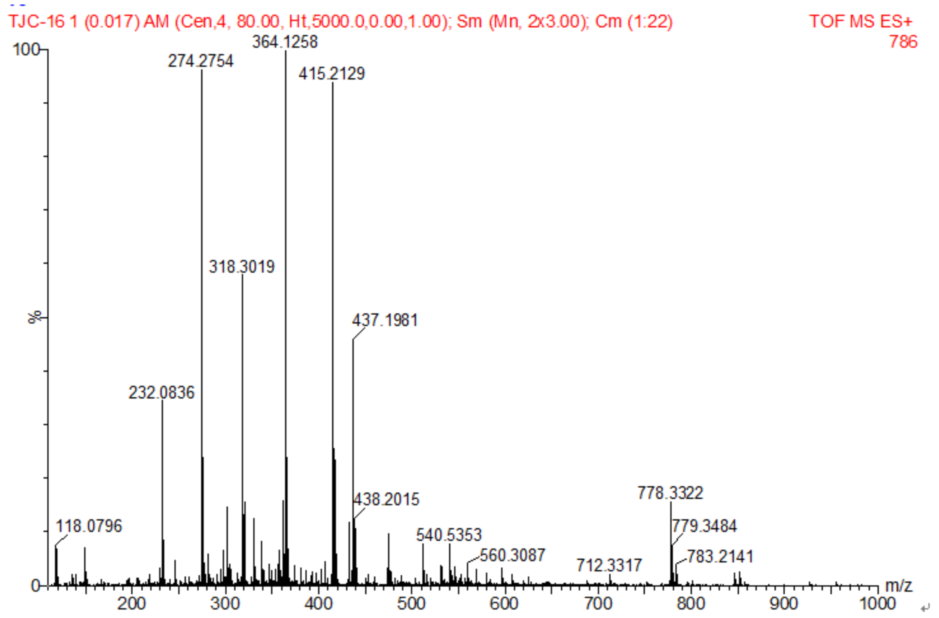


## Product 15: HRMS spectra

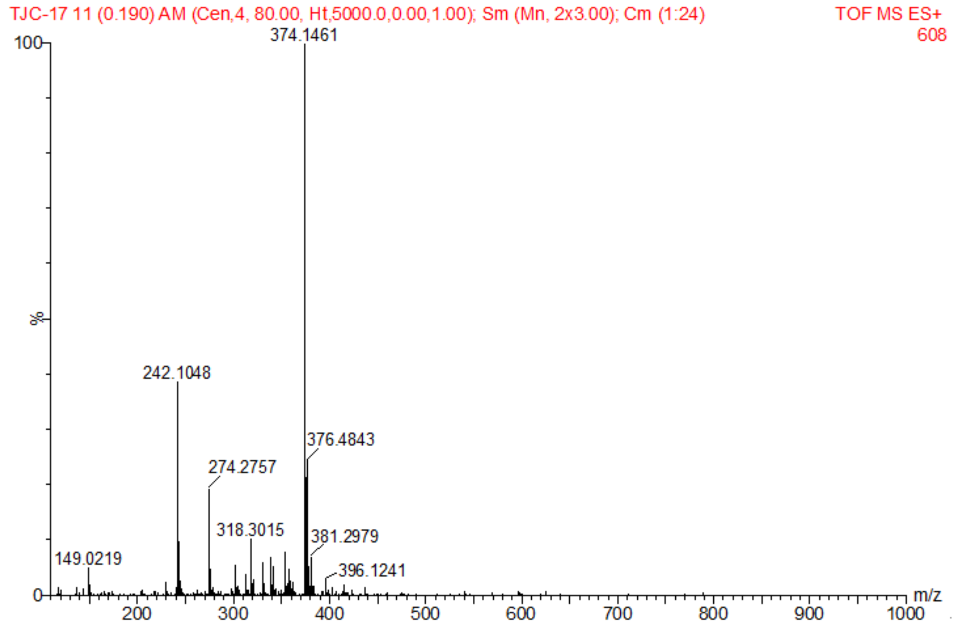

## Product 16: HRMS spectra

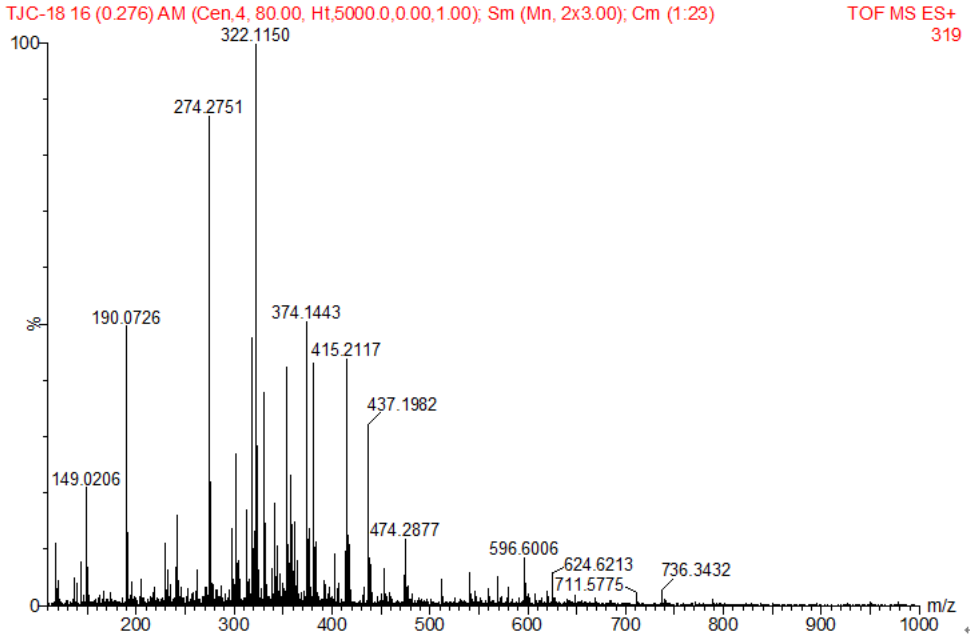

# Crystal data and structure refinement for compound 7

| | |
|---|---|
| Identification code | 201804211 |
| Empirical formula | $C_{10}H_{13}N_5O_5$ |
| Formula weight | 283.25 |
| Temperature/K | 293(2) |
| Crystal system | orthorhombic |
| Space group | $P2_12_12_1$ |
| a/Å | 4.7669(2) |
| b/Å | 11.0409(5) |
| c/Å | 21.6334(9) |
| α/° | 90 |
| β/° | 90 |
| γ/° | 90 |
| Volume/Å$^3$ | 1138.59(8) |
| Z | 4 |
| $\rho_{calc}$g/cm$^3$ | 1.652 |
| μ/mm$^{-1}$ | 1.157 |
| F(000) | 592.0 |
| Crystal size/mm$^3$ | 0.15 × 0.11 × 0.1 |
| Radiation | CuKα (λ = 1.54184) |
| 2Θ range for data collection/° | 8.174 to 134.138 |
| Index ranges | $-3 \leq h \leq 5$, $-13 \leq k \leq 13$, $-25 \leq l \leq 25$ |
| Reflections collected | 4100 |
| Independent reflections | 2032 [$R_{int}$ = 0.0304, $R_{sigma}$ = 0.0405] |
| Data/restraints/parameters | 2032/0/184 |
| Goodness-of-fit on $F^2$ | 1.036 |
| Final R indexes [I>=2σ (I)] | $R_1$ = 0.0366, $wR_2$ = 0.0880 |
| Final R indexes [all data] | $R_1$ = 0.0408, $wR_2$ = 0.0924 |
| Largest diff. peak/hole / e Å$^{-3}$ | 0.14/-0.21 |
| Flack parameter | 0.2(2) |

## Crystal data and structure refinement for compound 8

| | |
|---|---|
| Identification code | 201804210 |
| Empirical formula | $C_{10}H_{15}IN_6O_5$ |
| Formula weight | 426.18 |
| Temperature/K | 293(2) |
| Crystal system | monoclinic |
| Space group | $P2_1$ |
| a/Å | 5.08845(13) |
| b/Å | 12.7514(2) |
| c/Å | 11.3200(2) |
| α/° | 90 |
| β/° | 91.4146(19) |
| γ/° | 90 |
| Volume/Å$^3$ | 734.28(3) |
| Z | 2 |
| $\rho_{calc}$g/cm$^3$ | 1.928 |
| μ/mm$^{-1}$ | 17.478 |
| F(000) | 420.0 |
| Crystal size/mm$^3$ | 0.15 × 0.13 × 0.11 |
| Radiation | CuKα (λ = 1.54184) |
| 2Θ range for data collection/° | 7.812 to 134.094 |
| Index ranges | $-3 \leq h \leq 6, -15 \leq k \leq 15, -13 \leq l \leq 12$ |
| Reflections collected | 5309 |
| Independent reflections | 2616 [$R_{int}$ = 0.0300, $R_{sigma}$ = 0.0391] |
| Data/restraints/parameters | 2616/3/213 |
| Goodness-of-fit on $F^2$ | 1.062 |
| Final R indexes [I>=2σ (I)] | $R_1$ = 0.0286, $wR_2$ = 0.0701 |
| Final R indexes [all data] | $R_1$ = 0.0296, $wR_2$ = 0.0711 |
| Largest diff. peak/hole / e Å$^{-3}$ | 0.56/-0.62 |
| Flack parameter | -0.021(5) |
