# Supplementary figures and images for: Investigation of 8-Aza-7-Deaza Purine Nucleoside Derivatives
Source: Molecules. 2019 Mar 11;24(5):983. doi: 10.3390/molecules24050983 (PMC6429420; doi:10.3390/molecules24050983)

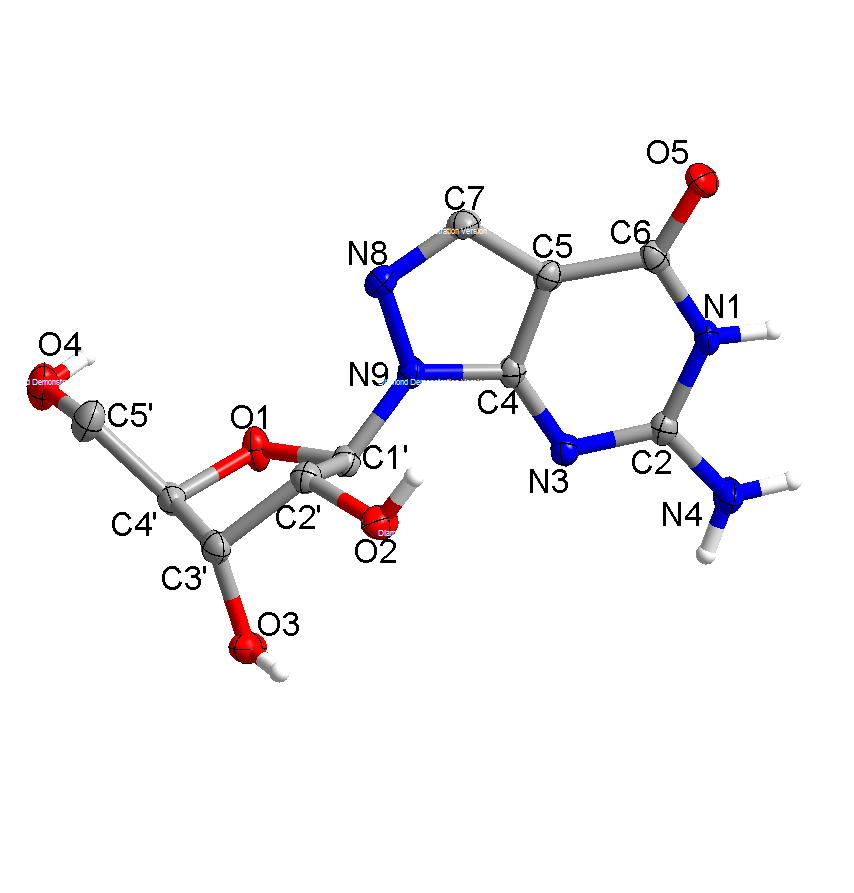

Supplement: Supplementary file 1 [file molecules-24-00983-s001.zip › molecules-460200 proof supple/supplementory materials Dr. An/cpd 7-Crystallographic information/201804211-1.bmp]

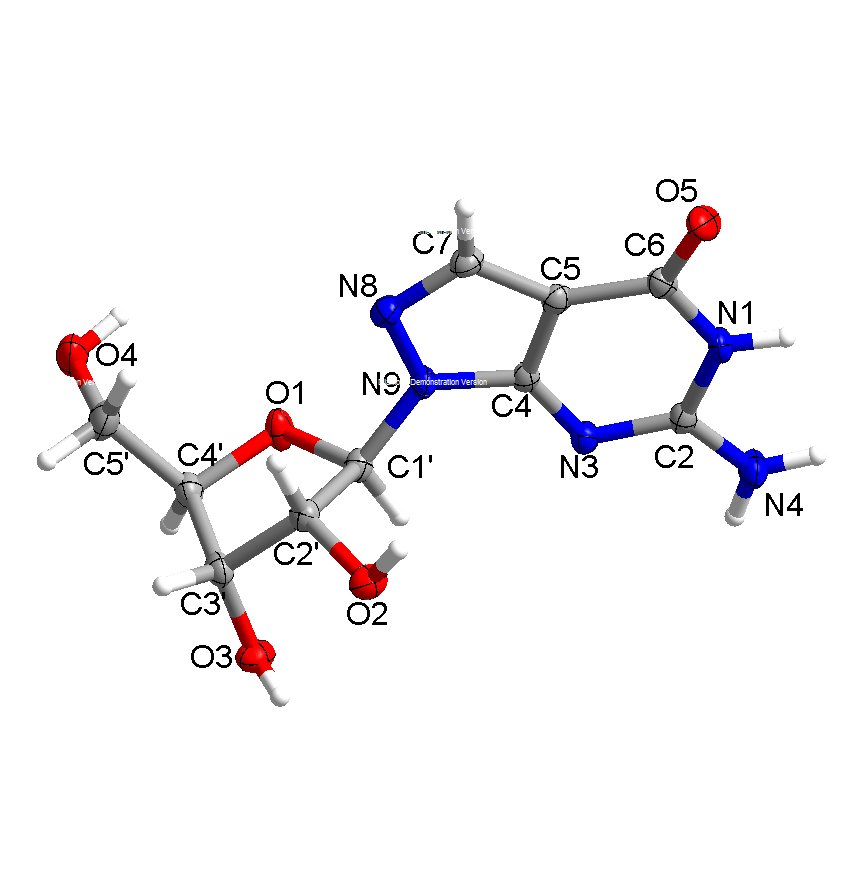

Supplement: Supplementary file 1 [file molecules-24-00983-s001.zip › molecules-460200 proof supple/supplementory materials Dr. An/cpd 7-Crystallographic information/201804211.bmp]

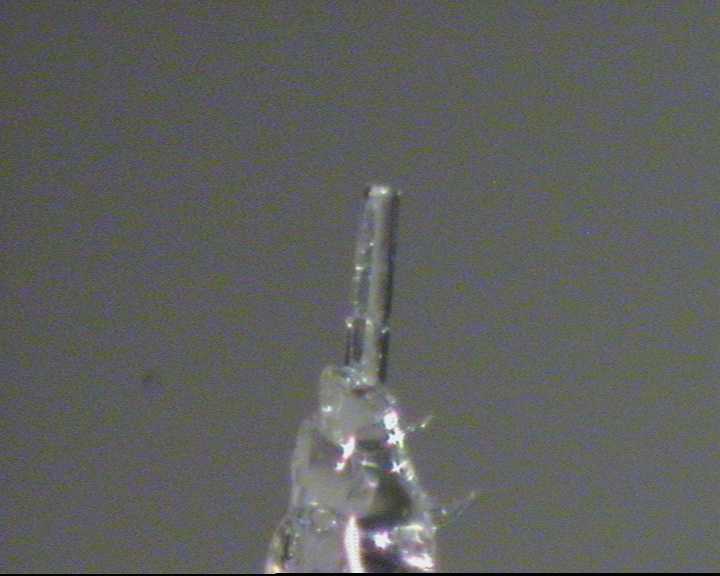

Supplement: Supplementary file 1 [file molecules-24-00983-s001.zip › molecules-460200 proof supple/supplementory materials Dr. An/cpd 7-Crystallographic information/20180421110.jpg]

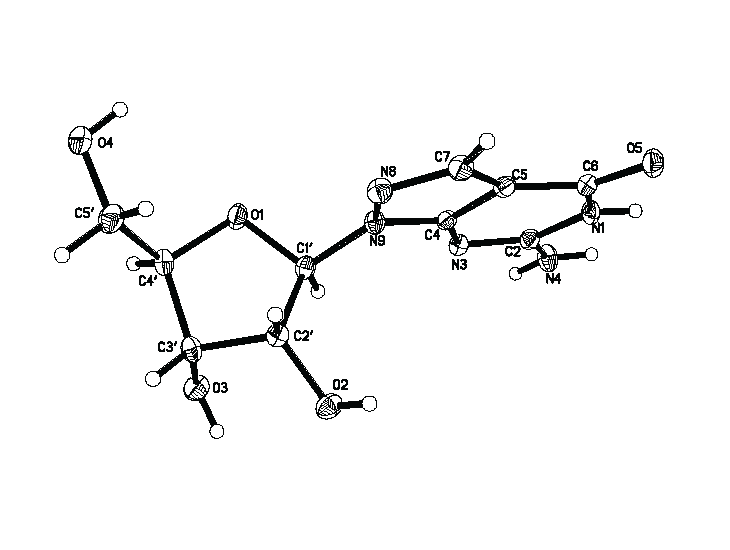

Supplement: Supplementary file 1 [file molecules-24-00983-s001.zip › molecules-460200 proof supple/supplementory materials Dr. An/cpd 7-Crystallographic information/p1.tif]

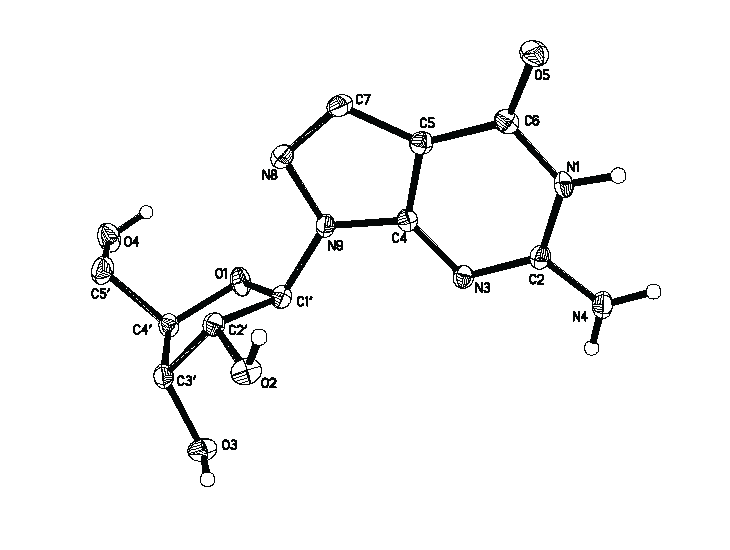

Supplement: Supplementary file 1 [file molecules-24-00983-s001.zip › molecules-460200 proof supple/supplementory materials Dr. An/cpd 7-Crystallographic information/p2.tif]

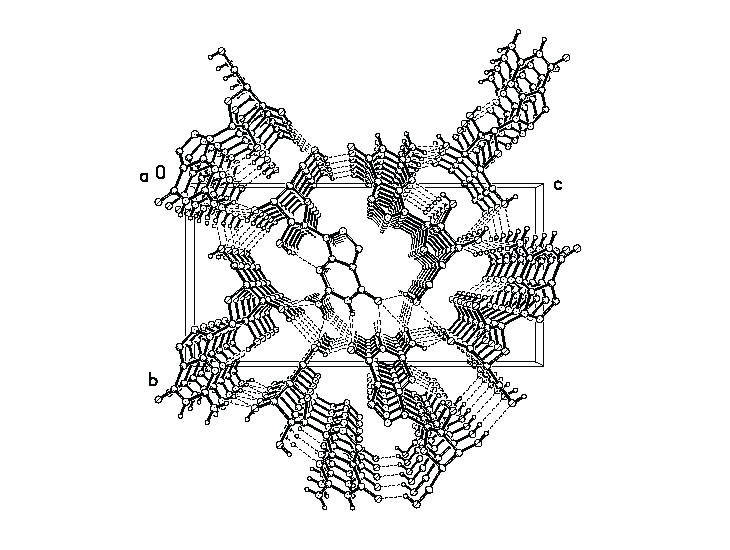

Supplement: Supplementary file 1 [file molecules-24-00983-s001.zip › molecules-460200 proof supple/supplementory materials Dr. An/cpd 7-Crystallographic information/p3.tif]

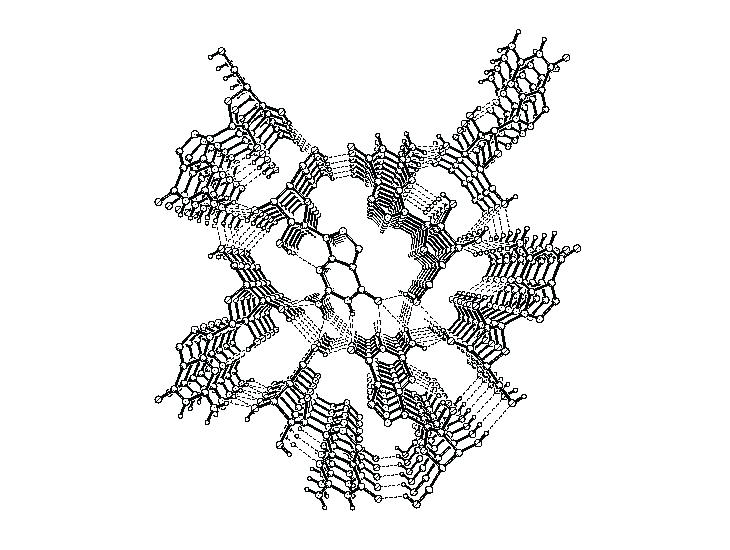

Supplement: Supplementary file 1 [file molecules-24-00983-s001.zip › molecules-460200 proof supple/supplementory materials Dr. An/cpd 7-Crystallographic information/p4.tif]

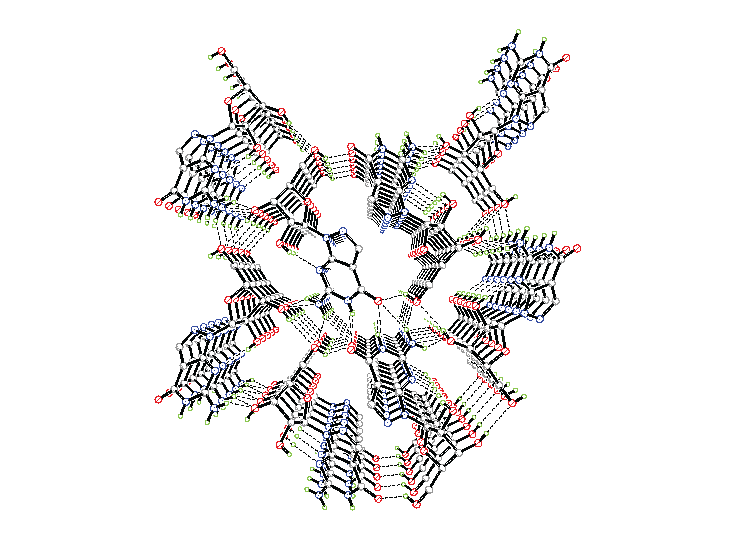

Supplement: Supplementary file 1 [file molecules-24-00983-s001.zip › molecules-460200 proof supple/supplementory materials Dr. An/cpd 7-Crystallographic information/p4a.tif]

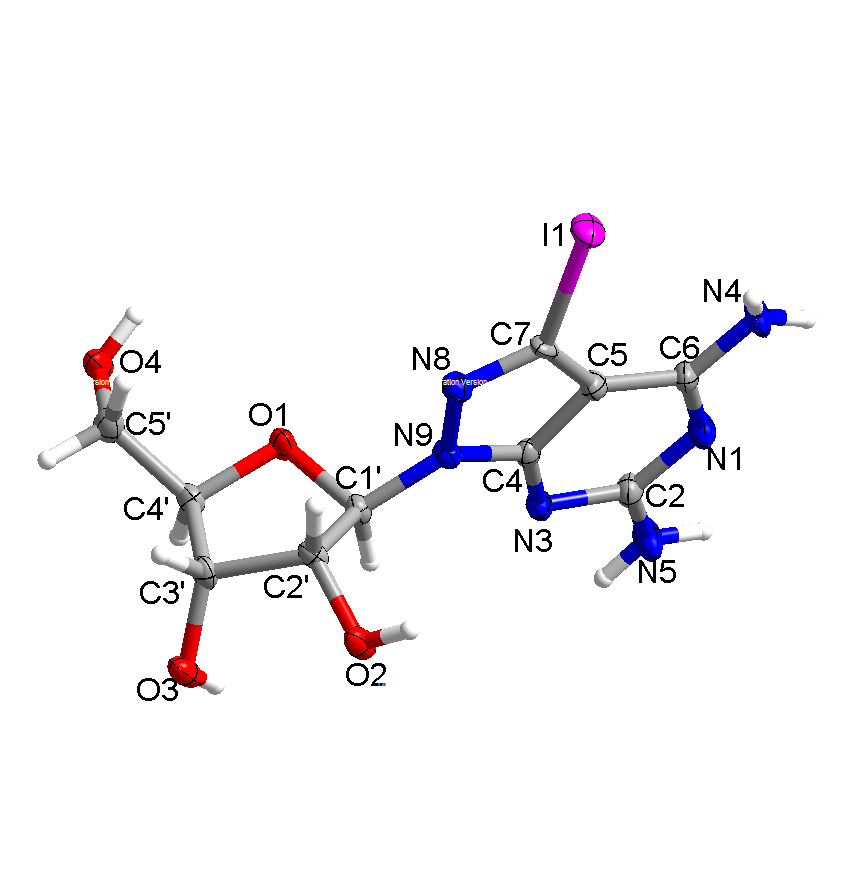

Supplement: Supplementary file 1 [file molecules-24-00983-s001.zip › molecules-460200 proof supple/supplementory materials Dr. An/cpd 8-Crystallographic information/201804210-1.bmp]

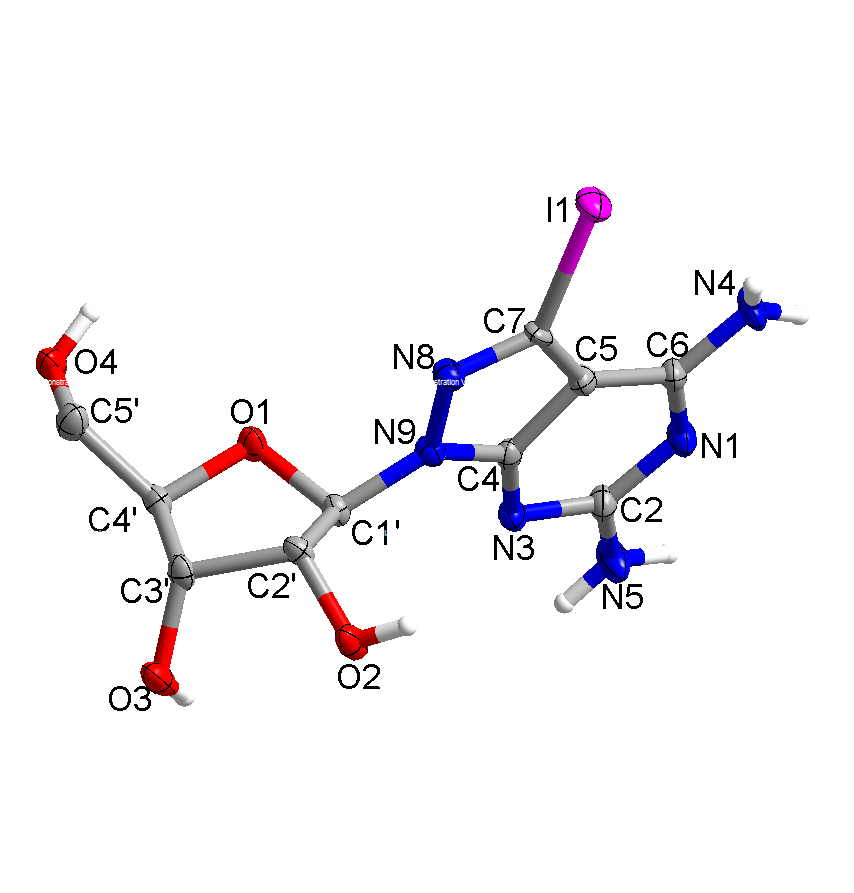

Supplement: Supplementary file 1 [file molecules-24-00983-s001.zip › molecules-460200 proof supple/supplementory materials Dr. An/cpd 8-Crystallographic information/201804210-2.bmp]

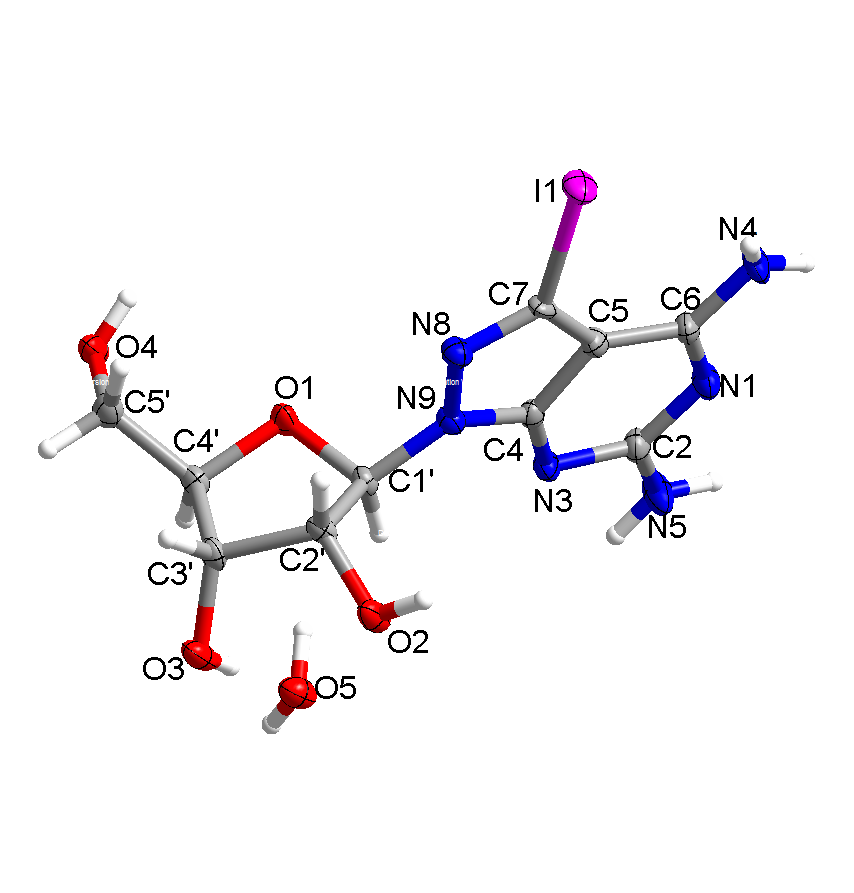

Supplement: Supplementary file 1 [file molecules-24-00983-s001.zip › molecules-460200 proof supple/supplementory materials Dr. An/cpd 8-Crystallographic information/201804210.bmp]

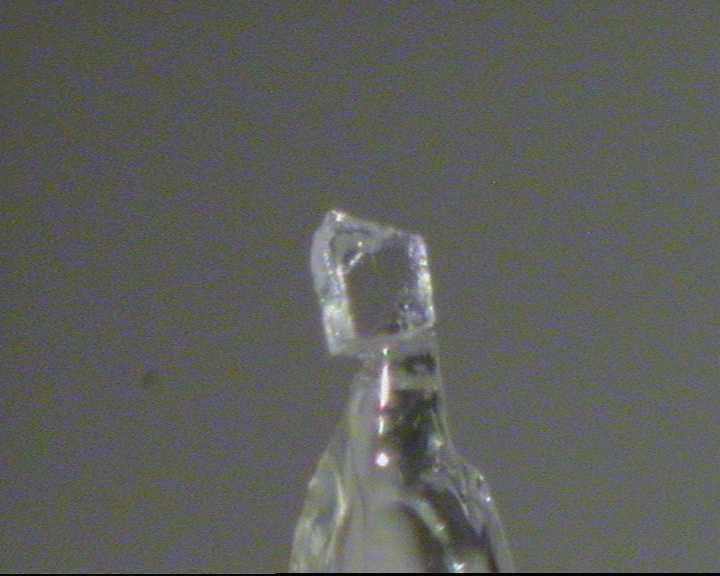

Supplement: Supplementary file 1 [file molecules-24-00983-s001.zip › molecules-460200 proof supple/supplementory materials Dr. An/cpd 8-Crystallographic information/2018042109.jpg]

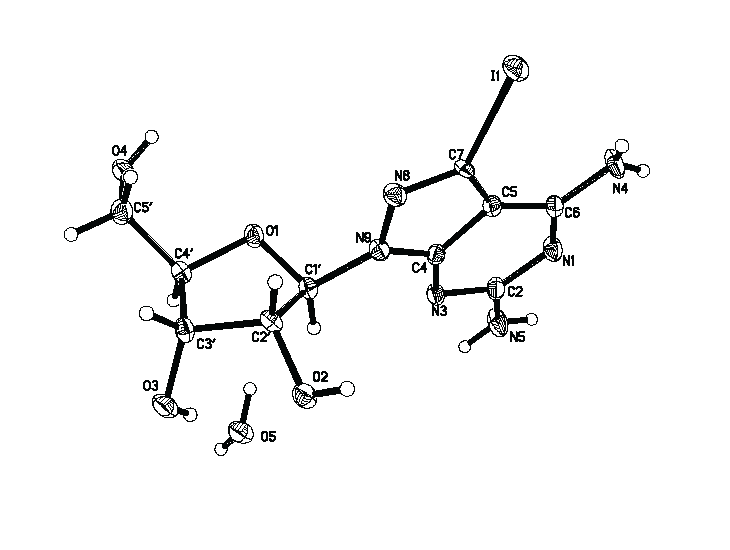

Supplement: Supplementary file 1 [file molecules-24-00983-s001.zip › molecules-460200 proof supple/supplementory materials Dr. An/cpd 8-Crystallographic information/p1.tif]

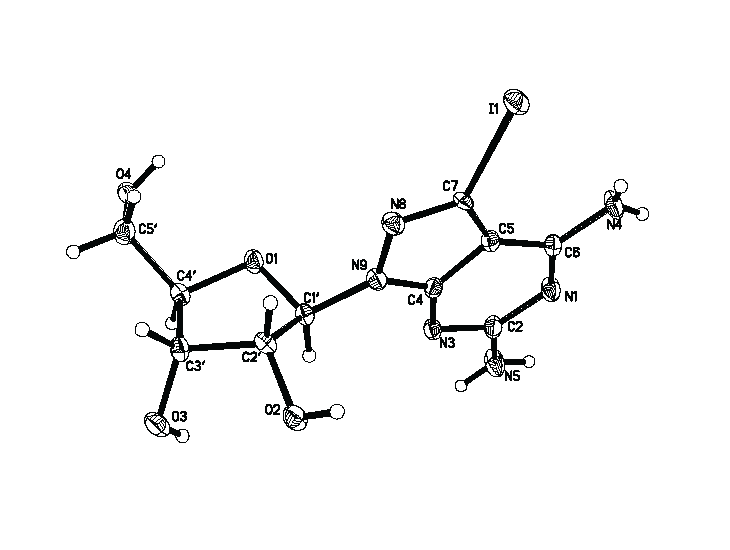

Supplement: Supplementary file 1 [file molecules-24-00983-s001.zip › molecules-460200 proof supple/supplementory materials Dr. An/cpd 8-Crystallographic information/p2.tif]

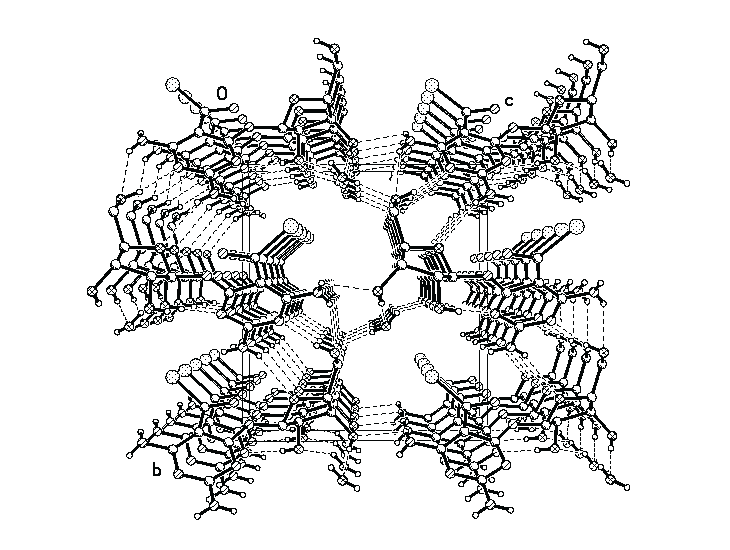

Supplement: Supplementary file 1 [file molecules-24-00983-s001.zip › molecules-460200 proof supple/supplementory materials Dr. An/cpd 8-Crystallographic information/p3.tif]

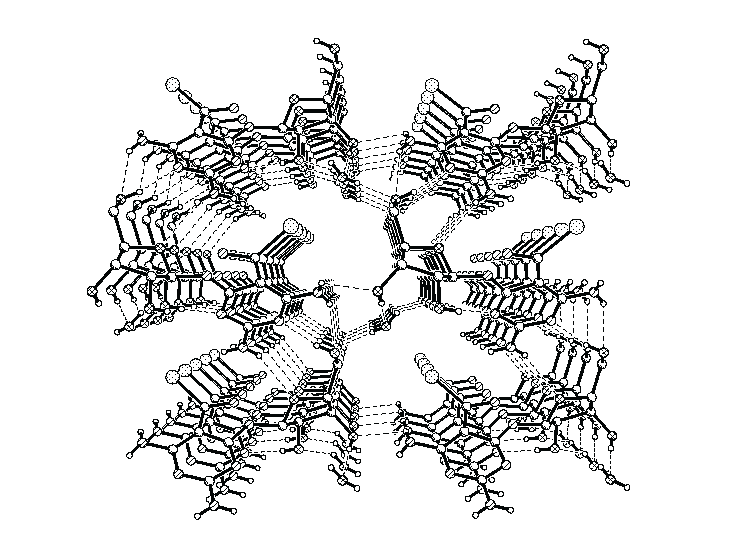

Supplement: Supplementary file 1 [file molecules-24-00983-s001.zip › molecules-460200 proof supple/supplementory materials Dr. An/cpd 8-Crystallographic information/p4.tif]

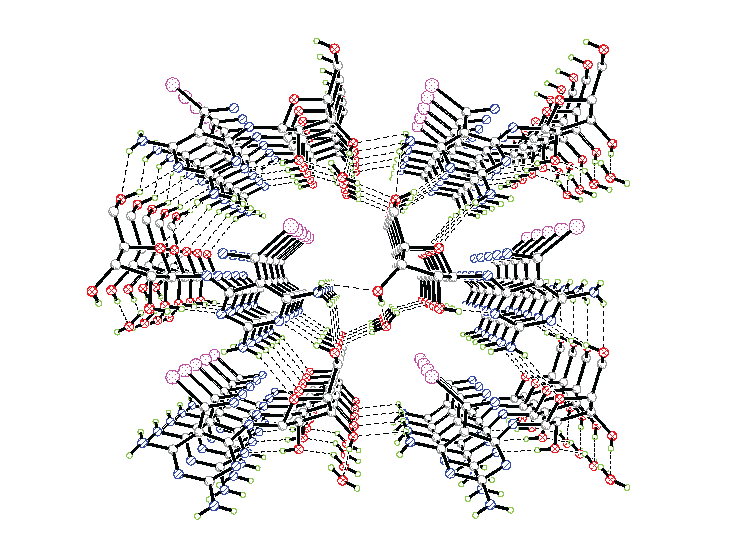

Supplement: Supplementary file 1 [file molecules-24-00983-s001.zip › molecules-460200 proof supple/supplementory materials Dr. An/cpd 8-Crystallographic information/p4a.tif]

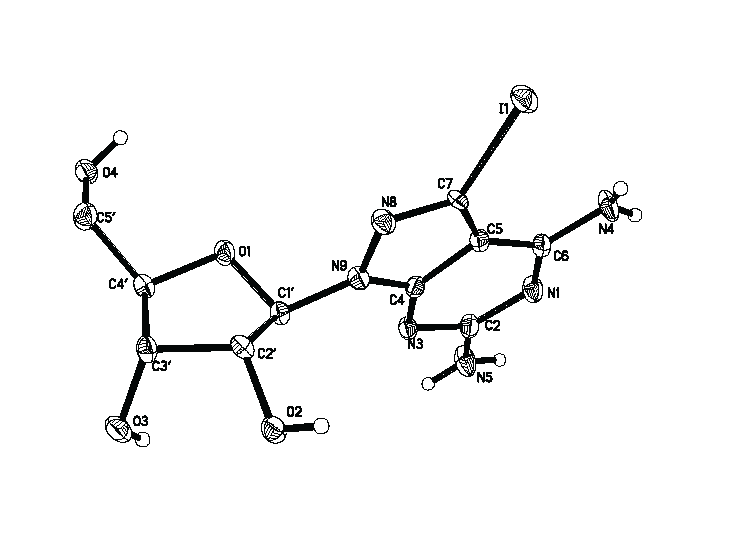

Supplement: Supplementary file 1 [file molecules-24-00983-s001.zip › molecules-460200 proof supple/supplementory materials Dr. An/cpd 8-Crystallographic information/p5.tif]
